# A comprehensive assessment of deworming coverage among pregnant women in low- and middle-income countries, 2000–30

**DOI:** 10.7189/jogh.14.04002

**Published:** 2024-03-01

**Authors:** Miho Sassa, Daisuke Yoneoka, Chris Fook Sheng Ng, Alton Quan Cao, Ganan Devanathan, Masahiro Hashizume, Shuhei Nomura

**Affiliations:** 1 Department of Global Health Policy, Graduate School of Medicine, The University of Tokyo, Tokyo, Japan; 2 Center for Surveillance, Immunization, and Epidemiologic Research, National Institute of Infectious Diseases, Tokyo, Japan; 3 Tokyo Foundation for Policy Research, Tokyo, Japan; 4 Department of Health Policy and Management, School of Medicine, Keio University, Tokyo, Japan

## Abstract

**Background:**

Intestinal parasitic infections pose a significant global public health issue, particularly among pregnant women, who are generally more susceptible due to their elevated need for iron and nutrients. Deworming stands as a secure and efficacious public health intervention. The World Health Organization (WHO) set a target for the national deworming coverage rate among pregnant women at 75% by 2030. Nonetheless, the existing body of evidence on deworming among pregnant women in low- and middle-income countries (LMICs) remains limited.

**Methods:**

Based on Demographic Health Survey (DHS) data from 56 LMICs (n = 924 277) between 2000 and 2022, we used Bayesian hierarchical models to estimate trends of deworming coverage up to 2030 and to analyse determinant factors of deworming.

**Results:**

We found that, despite progress in deworming coverage estimates for most countries, only 11 (<20%) are on track to achieve the WHO target coverage at the national level. Inequality gaps were projected to increase in most LMICs. A multilevel model showed that increased numbers of antenatal care, access to safe water, and a higher wealth index were associated with higher odds of deworming.

**Conclusions:**

The progress on deworming coverage and inequality in many countries remains insufficient for achieving the WHO target by 2030. Additional investments in the health sector towards the expansion of deworming programmes, along with integration with existing health services, are urgently required, as is the introduction of effective policies and strengthening programmes within the context of the ‘Leave No One Behind’ agenda.

Intestinal parasitic infections pose a significant global public health challenge. They are particularly prevalent in remote, rural areas where sanitation is insufficient and where environments are often contaminated with human and animal excreta [1]. The most common agents for these infections are soil-transmitted helminths and *schistosoma*, which caused 3.5 and 2.5 million disability-adjusted life years (DALYs) in 2016, respectively [2]. Intestinal parasites harm human health through various mechanisms, including blood loss and reduced absorption of nutrients and vitamins, leading to poor growth and delayed cognitive development; iron deficiency and anaemia; and undernutrition [3,4].

Pregnant women are substantially more prone to the effects of intestinal parasites due to their heightened susceptibility to conditions like anaemia and undernutrition, stemming from increased demands for iron and essential nutrients during both prenatal and postnatal periods [5]. For example, hookworm infection is a prominent contributor to anaemia among pregnant women in low- and middle-income countries (LMICs) [6-8]. Similarly, *schistosoma* infection has been linked to maternal anaemia [6]. The presence of anaemia in pregnant women increases the risks of adverse outcomes (e.g. maternal mortality and low birth weight) for both the mother and the newborn [9,10]. It is therefore widely acknowledged that women of reproductive age (WRA), including pregnant women, are significantly vulnerable to intestinal parasite infections, leading to significant morbidity. Per existing estimates, around 500 million WRA, including 138 million pregnant and lactating women, are at risk of soil-transmitted helminths and *schistosoma* infections, with an total DALYs of 0.68 million in 2015 [11,12].

The primary approach to controlling or eliminating soil-transmitted helminths and *schistosoma* infections is through preventive chemotherapy (PC), which involves the periodic mass drug administration of safe and quality-assured drugs and is recognised as one of the most effective public health strategies [11,12]. Despite pregnant women being particularly susceptible to the adverse impacts of intestinal parasitic infections, actions and initiatives aimed at this group, including PC, have been reserved. The World Health Organization (WHO) has recommended that pregnant women residing in areas endemic for soil-transmitted helminths and/or *schistosoma* should undergo deworming treatment after the first trimester of pregnancy. The overarching goal is to attain a national deworming coverage rate of 75% by 2030 [13]. However, the evidence that assesses the progress towards the achievement of this target is scarce. To the best of our knowledge, studies have yet to estimate trends or develop projections for deworming coverage among pregnant women based on existing research. Most of the available literature only estimated the coverage in specific areas at the national level [14,15]. Moreover, few studies assessed the change in inequality of deworming coverage among pregnant women. Defining the determinant factors for deworming help with informing policymakers in evaluating and strengthening such programmes and in designing new policies which will help in achieving the WHO target by 2030. Within the context of Universal Health Coverage (UHC), addressing disparities in vital health services, including deworming, is paramount. As such, an examination of historical trends and the evaluation of socioeconomic inequalities become indispensable in the process of monitoring advancements toward UHC. To bridge this information gap, a comprehensive evaluation of deworming coverage is a key priority.

## METHODS

### Country selection and study population

This study is a secondary analysis based on data from nationally representative household surveys conducted in LMICs since 2000. The study period from 2000 to 2030 is strategically chosen: The year 2000 serves as a baseline, coinciding with the start of significant global health initiatives which have influenced the prevention and control of intestinal parasitic infections, such as the Millennium Development Goals. Meanwhile, the year 2030 aligns with the Sustainable Development Goals’ timeline, providing a crucial benchmark for evaluating the progress and effectiveness of health policies and interventions over a 30-year span.

We used data from countries where the Demographic and Health Survey (DHS) provided information on the outcome variable. The included countries consist of those categorised as intestinal parasitosis endemic countries by the WHO [13] and those that reported deworming among pregnant women in the final DHS report. We classified them into income categories that are defined by the World Bank as of December 2022 [16].

The primary population of interest was women those who gave birth in the past five years prior to the time of the survey.

### Data source

We performed a population-based analysis of 106 publicly-available DHSs from 56 LMICs between 2000 and 2022 [17]. The DHS is a cross-sectional survey that offers nationally representative data with high response rates; it uses standardised methods and questionnaires to gather comprehensive information on various demographic and health-related topics relevant to LMICs [18] (**Online Supplementary Document**).

### Outcome variables of interest

Our primary outcome variable of interest was the coverage of deworming among pregnant women. In the DHS surveys, each mother was asked whether they took medicine for intestinal parasites during the last pregnancy of their last birth among those who gave birth in the past five years prior to the time of survey (‘During the last pregnancy, did you take any medicine for intestinal worms?’) [19]. We evaluated the outcome variable based on the pregnant women’s response.

### Predictor variables for the coverage projection

We selected relevant national-level predictor variables, the socio-demographic index (SDIs), the ratio of government health spending per gross domestic product (GDP), the ratio of access to safe water, the ratio of access to improved sanitation, the ratio of skilled health workforce (doctor, nurse, and midwife), and the GDP per capita following previous studies [20,21]. We used country- and year-specific variables to estimate the time trend and the projection of deworming coverage for each country. We obtained data on SDI, government health spending per GDP, access to safe water, and access to improved sanitation from the Global Health Data Exchange [22]; on the skilled health workforce from Global Health Observatory Data Repository hosted by the WHO [23]; and on the GDP per capita from the International Monetary Fund (IMF) [24].

### Determinant factors

We considered the availability, comparability, and consistency with previous literature [25–27] for a range of individual-, household-, and community-level independent variables such as age in years at the time of survey response (15–20, 20–24, 25–29, 30–34, 35–39, 40–44, and 45–49); religion (Christian, Muslim, and others); number of antenatal care (ANC) visits (none, one, two, three, and four or more times); media exposure (none, low, high); barrier to health care access (no or yes); access to the safe water (no or yes); access to adequate sanitation (no or yes); place of residence (urban or rural); maternal education level (no, primary, secondary, or higher); household wealth index (poorest, poorer, middle, richer, and richest), sex of household head (male or female); current marital status (not married or married); and given or bought iron tablets/syrup during last pregnancy (no or yes).

### Statistical analysis of coverage and equity

We estimated the deworming coverage for pregnant women using the sampling weights in the DHS survey data, stratified by wealth quintile and place of residence. To estimate the time trend of deworming coverage over study period, we set up Bayesian hierarchical linear regression models with three hierarchical structures: region, country, and year (**Online Supplementary Document**). To construct the final estimate up to 2030, we employed a two-step approach: first, each predictor explained in the above subsection was predicted up to 2030 using hierarchical linear regression with random intercept and slopes for time trend term. We then estimated the final hierarchical linear model based on the predicted predictors in the first step. We used a Markov chain Monte Carlo algorithm with two chains, 10 000 iterations, a thinning rate of 10, and 50% burn-in period. We estimated the 95% credible intervals (CrIs) for all indices using the 2.5th and 97.5th percentiles of the posterior distribution of the parameter. All models for national-level, across wealth quintile, and place of residence included the same covariates. We implemented Bayesian models with ‘JAGS 4.13’ in *R*, version 4.0.5. (R Core Team, Vienna, Austria).

We calculated the magnitude of wealth-based inequality in deworming coverage among pregnant women for each year using the slope index of inequality (SII), whose values range between −100 to +100, with the value equal to 0 indicating no inequality. We then estimated country-specific rate of change in coverage of deworming between 2000 and 2030 from the predicted values.

### Statistical analysis of determinant factors

We applied multivariable Bayesian multilevel logistic regression with random intercept country and region for the determinant analysis. Multilevel models account for the dependent nature of data across hierarchies, such as individuals nesting within countries and regions. Here we used multilevel models to examine whether variables differed by country and region based on the results of multicountry surveys. We performed the analysis in Stata, MP, version 17.0. (StataCorp LLC, College Station, TX, USA).

## RESULTS

### Survey characteristics

We included household survey data on 924 277 pregnant women from 56 LMICs based on 106 DHS data sets from January 2000 to December 2022. Deworming information among pregnant women was most commonly reported in Africa and Southeast Asia through DHS survey; meanwhile, coverage varied substantially across countries, from 0.6% in Armenia in 2010 to 85.8% in Congo in 2012 (Figure S1 and Table S1 in the **Online Supplementary Document**).

### National deworming coverage among pregnant women

**Figure 1:**
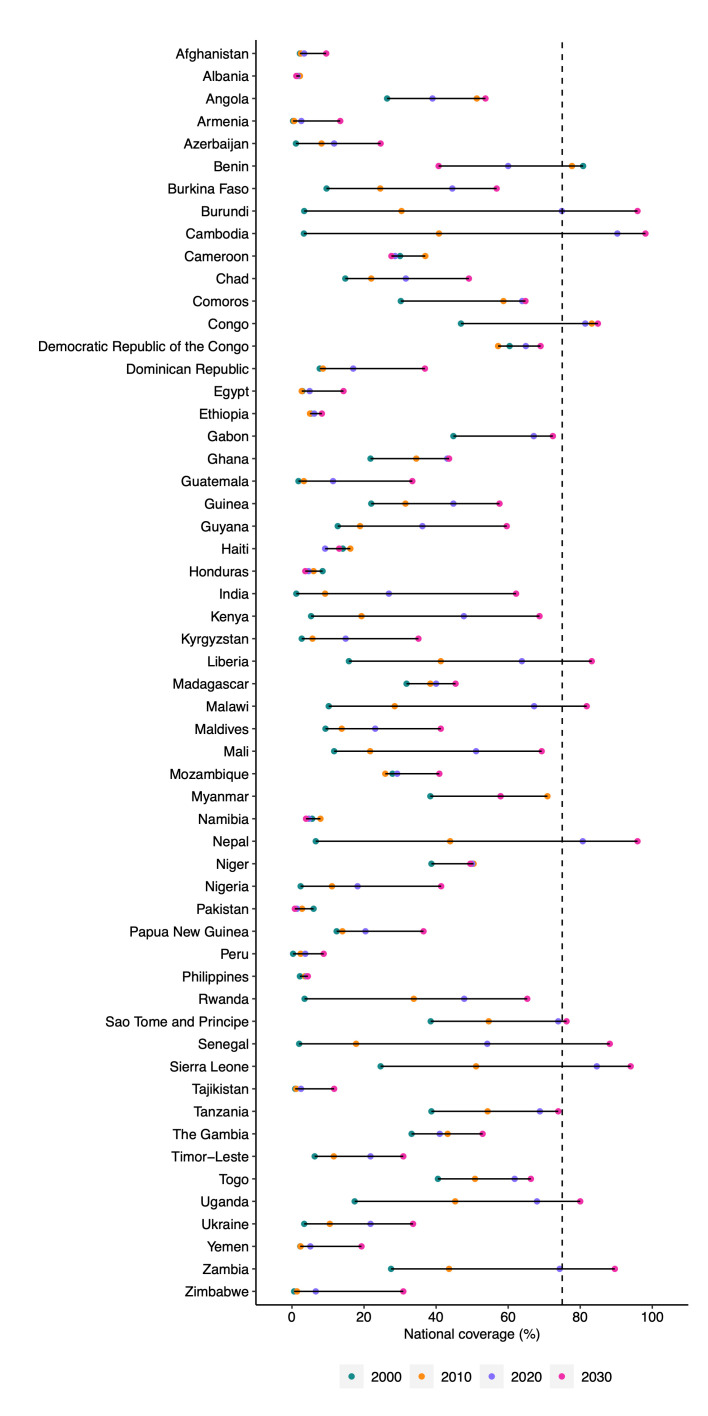
Estimated national coverage of deworming among pregnant women from 2000 to 2030. We estimated national deworming coverage among pregnant women in 2000, 2010, 2020, and 2030. Dotted line illustrates the WHO target deworming coverage among pregnant women by 2030.

Only four included LMICs (Cambodia, Congo, Nepal, and Sierra Leone) were predicted to achieve the WHO target of 75% coverage of deworming for pregnant women in 2020. Moreover, if the current trend continues, seven countries (Burundi, Liberia, Malawi, Sao Tome and Principe, Senegal, Uganda, and Zambia) were projected to achieve the target coverage by 2030 (**Figure 1**, **Table 1**).

**Table 1:** Estimated national coverage of deworming among pregnant women

	Coverage (95% credible intervals)			
**Country**	**2000**	**2010**	**2020**	**2030**
Afghanistan	2.2 (0.0–20.8)	2.5 (0.6–8.0)	3.4 (0.9–8.7)	9.5 (0.2–48.4)
Albania	2.1 (0.5–7.5)	2.2 (1.7–2.9)	1.6 (1.1–2.2)	1.2 (0.6–2.2)
Angola	26.4 (0.5–86.4)	51.3 (18.2–83.4)	39.0 (15.6–65.2)	53.7 (5.9–95.7)
Armenia	0.3 (0.0–1.7)	0.6 (0.5–0.9)	2.6 (0.3–11.0)	13.4 (0.1–74.9)
Azerbaijan	1.1 (0.1–4.4)	8.2 (2.1–21.2)	11.7 (0.6–48.2)	24.6 (0.2–92.7)
Benin	80.8 (58.1–94.5)	77.7 (70.9–83.7)	60.0 (44.5–73.8)	40.7 (13.2–73.4)
Burkina Faso	9.6 (0.5–38.8)	24.5 (19.2–30.5)	44.5 (7.7–89.1)	56.8 (2.7–99.0)
Burundi	3.4 (1.1–7.1)	30.4 (24.6–36.2)	74.9 (65.7–82.7)	95.9 (91.4–98.4)
Cambodia	3.3 (1.2–7.9)	40.8 (35.4–46.0)	90.3 (84.3–94.7)	98.1 (93.2–99.7)
Cameroon	30.0 (9.0–60.1)	37.0 (30.0–45.2)	28.6 (18.1–39.4)	27.6 (12.0–48.8)
Chad	14.8 (0.0–88.7)	22.0 (5.9–46.4)	31.6 (11.8–62.8)	49.1 (4.1–97.5)
Comoros	30.2 (2.2–83.3)	58.7 (46.7–70.3)	63.9 (29.4–88.8)	64.8 (7.3–98.5)
Congo	46.9 (3.9–94.5)	83.2 (71.1–91.3)	81.4 (47.0–97.0)	84.9 (24.0–99.8)
Democratic Republic of the Congo	60.4 (5.6–98.8)	57.2 (29.7–83.5)	64.9 (35.6–86.9)	69.0 (11.5–98.8)
Dominican Republic	7.7 (0.2–47.1)	8.6 (4.1–16.1)	17.0 (3.6–43.8)	36.9 (1.0–94.7)
Egypt	2.9 (0.1–17.5)	2.7 (1.1–5.4)	4.9 (1.3–12.6)	14.3 (0.3–65.1)
Ethiopia	5.3 (0.0–43.1)	5.0 (2.7–7.8)	6.2 (3.0–11.2)	8.3 (0.5–35.9)
Gabon	44.8 (4.3–92.5)	67.1 (53.3–78.8)	67.1 (32.8–90.8)	72.4 (13.1–99.3)
Ghana	21.8 (2.6–65.6)	34.5 (22.8–48.8)	43.1 (28.3–58.0)	43.6 (19.4–69.7)
Guatemala	1.8 (0.0–11.2)	3.3 (1.0–7.7)	11.4 (4.0–25.5)	33.4 (2.1–88.1)
Guinea	22.0 (6.3–53.1)	31.5 (21.3–42.4)	44.8 (35.7–54.7)	57.6 (33.5–81.2)
Guyana	12.7 (0.6–55.5)	18.9 (12.7–26.3)	36.2 (2.6–89.1)	59.6 (0.8–99.8)
Haiti	14.1 (5.5–26.0)	16.2 (9.0–25.3)	9.2 (6.1–12.8)	13.1 (6.3–22.2)
Honduras	8.5 (4.7–13.9)	6.0 (4.2–8.0)	4.6 (2.1–9.0)	3.7 (0.8–10.5)
India	1.2 (0.5–2.3)	9.2 (6.2–12.5)	26.9 (18.9–35.8)	62.2 (43.6–78.5)
Kenya	5.3 (2.1–10.5)	19.3 (15.2–23.6)	47.7 (32.9–61.4)	68.7 (37.4–90.1)
Kyrgyzstan	2.7 (0.0–18.4)	5.7 (2.7–10.3)	14.9 (2.7–42.3)	35.1 (1.1–93.4)
Liberia	15.8 (10.4–21.8)	41.3 (29.8–52.3)	63.8 (57.0–70.2)	83.2 (69.4–92.5)
Madagascar	31.8 (4.4–76.1)	38.4 (27.5–49.4)	40.0 (2.6–92.0)	45.4 (0.4–98.8)
Malawi	10.2 (0.8–41.8)	28.5 (22.7–35.3)	67.2 (46.4–83.3)	81.8 (39.2–98.6)
Maldives	9.3 (0.4–40.9)	13.8 (9.2–19.7)	23.1 (2.4–70.1)	41.3 (0.8–98.0)
Mali	11.7 (0.4–74.1)	21.7 (13.1–34.4)	51.1 (38.8–63.0)	69.3 (23.4–94.0)
Mozambique	27.9 (0.2–97.6)	25.9 (17.0–36.1)	29.2 (4.3–73.3)	40.9 (1.4–96.7)
Myanmar	38.4 (0.3–98.4)	70.9 (4.6–99.8)	57.9 (34.1–79.2)	57.9 (8.9–96.7)
Namibia	5.6 (2.2–14.3)	7.9 (5.7–10.4)	4.7 (1.8–12.5)	3.9 (0.6–15.5)
Nepal	6.6 (4.1–9.5)	43.9 (38.2–50.0)	80.7 (70.7–88.4)	95.9 (91.1–98.3)
Niger	38.7 (3.2–86.7)	50.4 (35.8–65.0)	50.0 (16.4–85.7)	49.5 (2.7–97.5)
Nigeria	2.4 (0.3–11.2)	11.1 (8.7–13.7)	18.2 (14.0–23.6)	41.4 (15.2–74.6)
Pakistan	6.0 (0.4–26.9)	2.8 (1.7–4.6)	1.4 (0.8–2.4)	0.8 (0.2–2.1)
Papua New Guinea	12.4 (0.0–82.4)	14.0 (1.7–48.1)	20.4 (12.3–30.3)	36.5 (2.9–86.6)
Peru	0.3 (0.1–0.6)	2.4 (2.0–2.9)	3.7 (1.0–8.7)	8.8 (1.3–28.4)
Philippines	2.2 (1.0–4.1)	3.6 (2.7–4.7)	4.1 (2.7–5.9)	4.4 (1.9–8.3)
Rwanda	3.5 (0.5–10.5)	33.8 (27.7–40.0)	47.8 (41.6–54.1)	65.3 (53.0–76.7)
Sao Tome and Principe	38.5 (4.1–87.2)	54.6 (42.0–67.2)	73.9 (19.7–97.9)	76.2 (4.9–99.8)
Senegal	2.0 (0.7–4.1)	17.8 (14.7–21.3)	54.2 (48.9–59.1)	88.2 (80.2–93.6)
Sierra Leone	24.6 (1.8–76.9)	51.1 (44.7–57.8)	84.6 (80.2–88.5)	94.0 (85.6–98.1)
Tajikistan	0.9 (0.0–6.6)	1.2 (0.2–4.2)	2.5 (1.1–5.1)	11.7 (0.4–53.5)
Tanzania	38.7 (1.4–94.0)	54.3 (23.4–80.8)	68.8 (48.0–84.1)	73.9 (18.8–98.4)
The Gambia	33.2 (4.7–76.7)	43.2 (31.1–55.6)	41.0 (33.6–48.5)	52.9 (21.2–83.5)
Timor-Leste	6.3 (0.2–33.9)	11.6 (9.0–15.0)	21.8 (8.7–43.7)	30.9 (2.9–87.2)
Togo	40.5 (1.0–96.7)	50.8 (28.6–70.5)	61.8 (31.1–87.7)	66.3 (9.9–98.7)
Uganda	17.4 (5.0–52.7)	45.3 (39.6–50.8)	68.0 (57.0–77.3)	80.0 (46.2–96.5)
Ukraine	3.4 (0.2–18.1)	10.5 (5.4–18.0)	21.8 (1.2–71.3)	33.6 (0.2–96.9)
Yemen	2.3 (0.0–17.9)	2.4 (0.9–5.1)	5.1 (0.2–25.7)	19.3 (0.2–85.5)
Zambia	27.5 (1.0–93.5)	43.6 (15.5–69.1)	74.3 (62.2–85.0)	89.6 (70.2–97.6)
Zimbabwe	0.6 (0.1–1.8)	1.4 (0.8–2.4)	6.6 (3.3–11.0)	30.9 (5.8–69.0)

### Urban-rural disparities in deworming coverage among pregnant women

**Figure 2:**
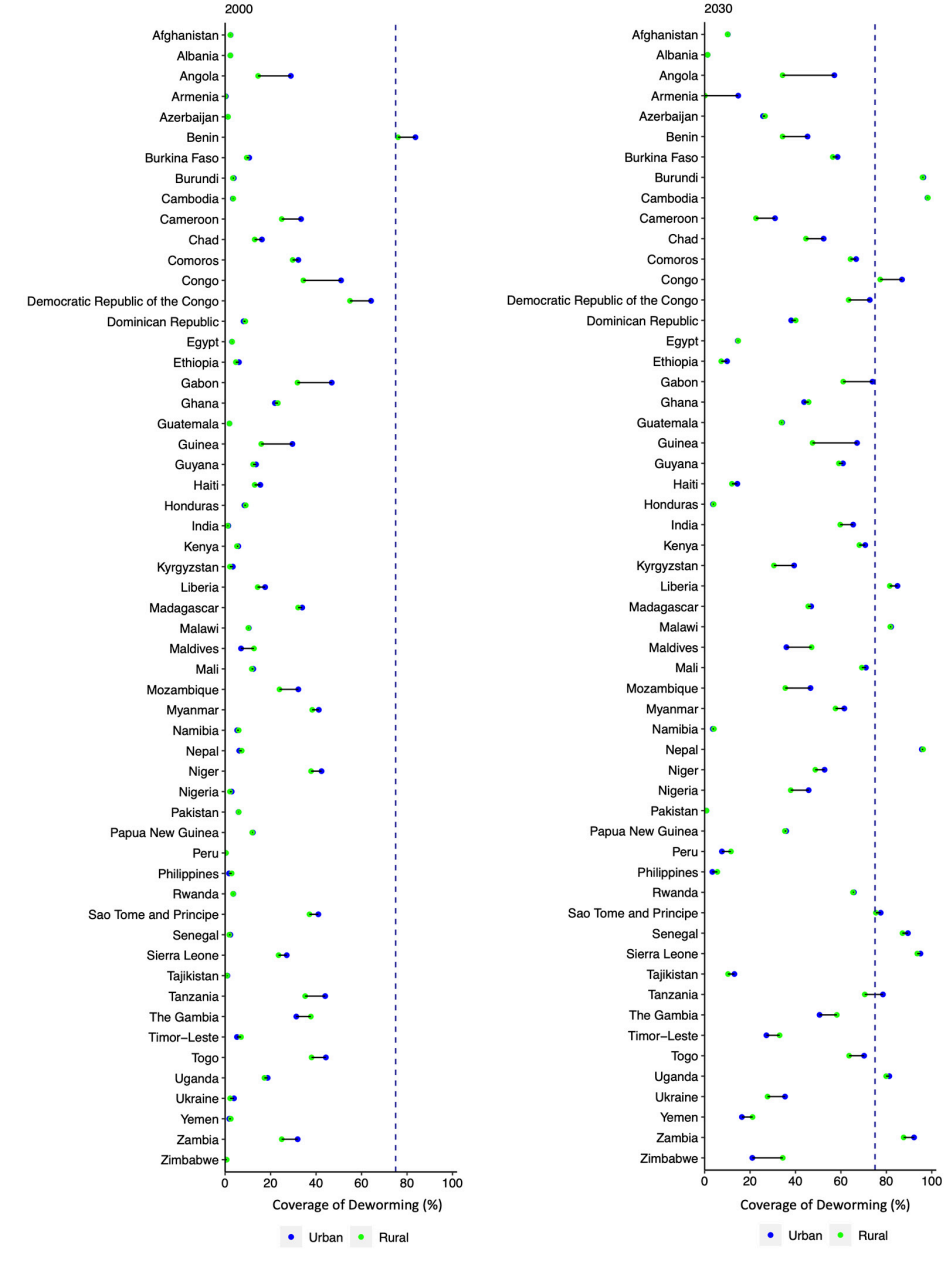
Coverage of deworming among pregnant women across place of residence, 2000–2030. We estimated national deworming coverage among pregnant women across place of residence (urban vs rural) in 2000 and 2030. Dotted line illustrates the WHO target deworming coverage among pregnant women by 2030.

Eleven countries (Burundi, Cambodia, Congo, Liberia, Malawi, Nepal, Sao Tome and Principe, Senegal, Sierra Leone, Uganda, and Zimbabwe) were projected to achieve the WHO target by 2030 for both urban and rural groups. In almost all countries, individuals who lived in urban areas had a higher probability of deworming during the last pregnancy compared to those in rural areas. In Tanzania, for example, pregnant women who live in urban areas are estimated to achieve the WHO target coverage by 2030, while pregnant women in rural areas will not achieve this target. The urban-rural disparities were estimated to increase in most of the countries from 2000 to 2030, with the highest projected difference in coverage between the groups in Angola at 22.8%, followed by 19.6% in Guinea and 14.7% in Armenia in 2030 (**Figure 2**; Figure S2 and Table S2 in the **Online Supplementary Document**).

### Wealth-based inequity in coverage of deworming among pregnant women

**Figure 3:**
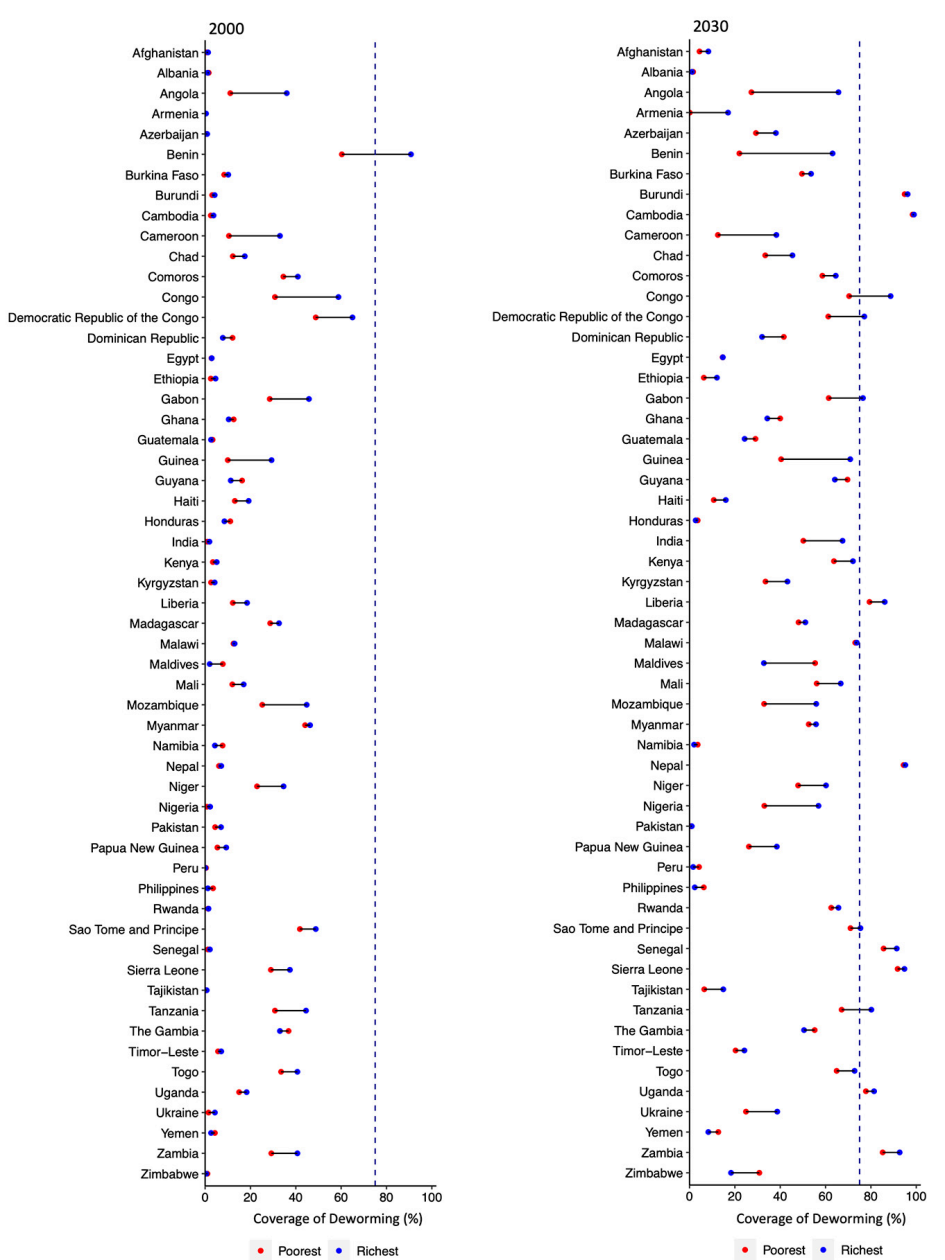
Coverage of deworming among pregnant women by wealth quintile, 2000–2030. We estimated national deworming coverage among pregnant women across wealth quintile (poorest vs richest) in 2000 and 2030. Dotted line illustrates the WHO target deworming coverage among pregnant women by 2030.

We assessed deworming coverage by household wealth quintiles to determine the magnitude of inequalities in deworming among pregnant women (**Figure 3**). Thirteen countries within the richest group were predicted to achieve the target coverage by 2030. However, among these countries, the coverage of the poorest quintile is projected to fail meeting the target in five countries.

Our estimates show that within-country disparity for most of the countries will increase from 2000 to 2030 (**Figure 3**). Although the coverage of deworming among pregnant women is generally estimated to improve in most of the LMICs, the individuals in the poorest quintile will have lower coverage than those in the richest quintile. The magnitude of socioeconomic inequality in the coverage of deworming among pregnant women is estimated to increase in most countries (Figure S3 and Table S3–4 in the **Online Supplementary Document**), and will be the greatest in Benin in 2030 (SII = 47.4), followed by Angola (SII = 42.7) and Guinea (SII = 37.3).

**Figure 4:**
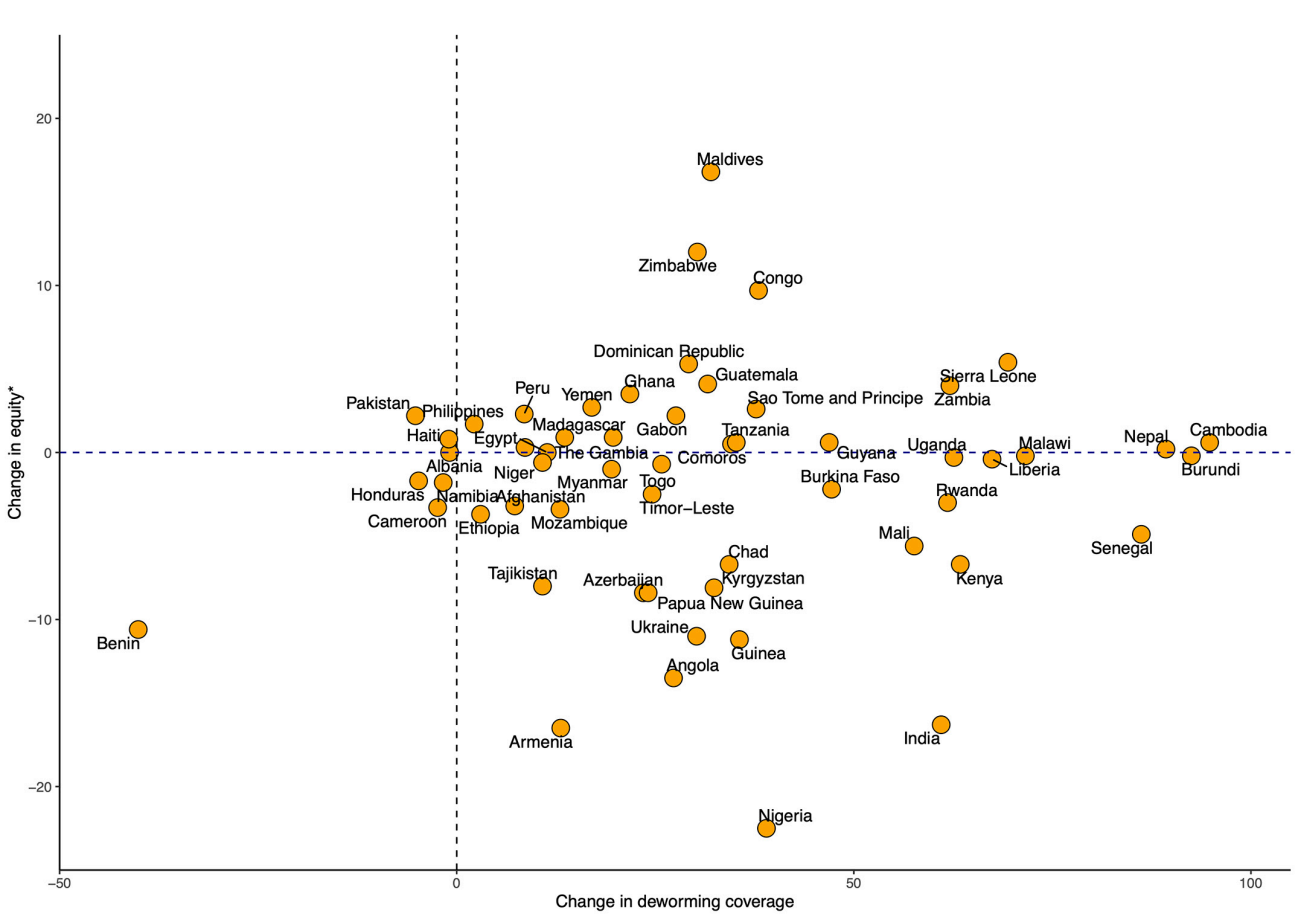
Trends in deworming coverage equity among pregnant women from 2000 to 2030. We estimated the discrete change in deworming coverage from 2000 to 2030 and the corresponding change in equity (poorest vs richest) for study countries. Change in equity: ((richest − poorest) of 2000) − ((richest − poorest) of 2030) (%).

Although most countries are estimated to improve deworming coverage at the national level, the inequality by socioeconomic status is projected to exacerbate in most countries (**Figure 4**). For example, although national coverage of deworming among pregnant women in India is predicted to increase by 2030, inequality by socioeconomic status will likely increase as well.

### Determinants of deworming among pregnant women

Based on latest DHS data, we included 471 930 individuals in the determinant analysis after dropping samples that had missing values of variables. Belonging to the ≥20-year-old age group was associated with higher odds of deworming. Increased rounds of ANC, having access to safe water (odds ratio (OR) = 1.08; 95% CrI = 1.07–1.09), living in rural areas (OR = 1.01; 95% CrI = 1.00–1.01), higher wealth index, and the provision/purchase of iron tablets/syrup (OR = 3.11; 95% CrI = 3.09–3.12) were associated with higher odds of deworming during the last pregnancy. Women of Muslim faith had lower odds of deworming (OR = 0.84; 95% CrI = 0.82–0.85) compared to women of Christian faith. Higher education level, a female household head, and unmarried women were associated with lower odds of deworming among pregnant women (**Table 2**).

**Table 2:** Determinant factors of deworming among pregnant women in 56 low– and middle–income countries

Characteristics	OR (95% CrIs)
Age	
*15–19*	1.00
*20–24*	1.05 (1.04*–*1.06)
*25–29*	1.07 (1.06*–*1.08)
*30–34*	1.02 (1.02*–*1.03)
*35–39*	1.04 (1.04*–*1.05)
*40–44*	1.01 (1.01*–*1.03)
*45–49*	1.12 (1.10*–*1.14)
Religion	
*Christian*	1.00
*Muslim*	0.88 (0.88*–*0.89)
*Other*	1.27 (1.26*–*1.28)
Number of ANC visits	
*None*	1.00
*1*	2.88 (2.86*–*2.89)
*2*	3.35 (3.34*–*3.35)
*3*	3.48 (3.47*–*3.50)
*≥4*	4.50 (4.47*–*4.54)
Media exposure level	
*None*	1.00
*Low*	1.03 (1.03*–*1.04)
*High*	1.17 (1.16*–*1.19)
Barrier to health care access	
*None*	1.00
*Yes*	1.31 (1.31*–*1.32)
Access to safe water	
*No*	1.00
*Yes*	1.08 (1.07*–*1.09)
Access to adequate sanitation	
*No*	1.00
*Yes*	0.98 (0.97*–*0.98)
Place of residence	
*Urban*	1.00
*Rural*	1.01 (1.00*–*1.01)
Maternal education level	
*No*	1.00
*Primary*	0.98 (0.98*–*0.98)
*Secondary*	0.93 (0.93*–*0.93)
*Higher*	0.76 (0.75*–*0.76)
Wealth index	
*Poorest*	1.00
*Poorer*	1.19 (1.18*–*1.20)
*Middle*	1.22 (1.22*–*1.23)
*Richer*	1.22 (1.20*–*1.23)
*Richest*	1.34 (1.33*–*1.35)
Sex of household head	
*Male*	1.00
*Female*	0.99 (0.98*–*1.00)
Current marital status	
*Not married*	1.00 (0.97*–*0.99)
*Married*	0.98
Given or bought iron tablets/syrup	
*No*	1.00
*Yes*	3.11 (3.09*–*3.12)

ANC – antenatal care, CrIs – credible intervals, OR – odds ratio

### Sensitivity analysis

By altering the prior distribution, the median absolute difference between the two sets of estimated results of deworming coverage among pregnant women was very small: 0.4% in 2000, 0.0% in 2010, −0.5% in 2020, and 0.0% in 2030. The posterior mean difference between the results was also small: 1.02% in 2000, 0.13% in 2010, −0.38% in 2020, and −0.72% in 2030. The deviance information criterion (DIC) scores of the models did not change after altering priori distribution (DIC = 182.1 (flat prior) vs 182.8 (weekly prior)). The potential scale reduction factors (PSRF) values for the model diagnostics indicated that estimated values and the upper limit for the model of deworming coverage among pregnant women were close to 1 (Table S5–6 in the **Online Supplementary Document**).

## DISCUSSION

We comprehensively analysed the deworming coverage among pregnant women in LMICs based on nationally representative survey data from 56 countries between 2000 and 2022, covering approximately one million individuals. We found a positive trend in deworming coverage among pregnant women in most countries from 2000 to 2030. Although there was an overall increase in coverage, we observed significant disparities both within and between countries.

We found that only 11 out of 56 included LMICs are on track to achieve the WHO target for deworming coverage of 75% at the national level by 2030. Countries from sub-Saharan Africa were predicted to have higher coverage of deworming, which is consistent with the result for deworming in children [28]. This could be explained by the fact that programmes of neglected tropical diseases (NTDs) have been focussed on Africa due to endemicity and associated morbidities. As the importance of deworming for WRA has been emphasised, and with accumulating evidence on efficacy, safety, and outcomes of deworming during pregnancy, the WHO added women of child-bearing age as a target of PC in the new roadmap to 2030 [13]. Definitive action is needed to ensure that WRA are included in national programmes and policies. Deworming is one of the most cost-effective public health interventions for this purpose, and can be very affordable if integrated with existing health care services such as ANC [29].

Besides estimating deworming coverage among pregnant women, we also assessed levels of inequalities based on socioeconomic and geographical factors. Despite the projected progress of deworming coverage for most countries, the inequality gap will largely remain or expand by 2030 in most LMICs. Here we estimated a wide wealth-based gap in Angola, Benin, Cameroon, Guinea, Mozambique, and Nigeria, in contrast to previous studies that assessed maternal health service inequality such as ANC and facility delivery [21]. This may be due to the nature of endemicity of intestinal parasitosis, which is generally more prevalent among people who live in rural areas with poor access to sanitation and safe water, and who are likely to also have barriers to access to health care services. Countries have heterogeneous approaches to tackle disparities, especially socioeconomic-based inequality, some of which have been demonstrated to be sustainable. For example, Ghana is making great progress in improving wealth-based inequalities through implementing the National Health Insurance Scheme (NHIS), which enables pregnant women to be exempt from premiums via ensuring the coverage of maternity care [30], which may have resulted in a great decrease of absolute inequality from 2000 to 2020. Cambodia, meanwhile, succeeded in reducing inequality by socioeconomic factors, which has been attributed to several initiatives such as the ‘health equity funds’ and ‘community-based health insurance’ schemes that cover health care expenses for the poorest population [31].

We found that a higher number of ANC visits was associated with higher odds of deworming among pregnant women, implying that they play a great role in providing an opportunity to receive medicine for intestinal parasites during pregnancy. This is consistent with a previous study that used DHS data from sub-Saharan Africa [14]. Repeated ANC visits can help mothers by not only allowing them to receive health care and gaining knowledge, but also through developing rapport with their health care provider, resulting in better maternal and neonatal outcomes [32]. Mothers who were provided or purchased iron tablets or syrup themselves showed higher odds of deworming during the last pregnancy. These results may reflect the availability and access to the necessary medicine during pregnancy of individuals, since iron is one of the most essential medicines for pregnant women [33].

We also observed that deworming coverage was higher with increased media exposure, which is the consistent with results from a previous study [14]. Media has great influence on access and utilisation of maternal health services, especially for pregnant women, who generally rely on correct information to seek and access appropriate health care services with appropriate timing. They can aid pregnant women greatly by disseminating health-related information and helping mothers develop knowledge through visual appeals and audio messages which can easily reach and be understood by those who live in rural areas or have a low educational level. Public health policies and practices need to be aware and consider appropriate media sources to distribute information effectively in order to increase the uptake of deworming medication among pregnant women.

### Limitations

This study has some limitations. First, the outcome variable –deworming during the last pregnancy – was self-reported by the mothers and is susceptible to recall bias, a factor that may potentially fluctuate based on maternal wealth. Recall bias might also be present for mothers who lived in low-transmission settings, where mothers are less aware of the need for deworming medicine, resulting in them being less likely to recall receiving the medicines during the last pregnancy. However, some studies validated that the level of recall bias is relatively lower for the indicators of maternal health service [34]. Second, DHS survey data on deworming coverage among pregnant women from 46 intestinal parasitosis endemic countries was not available, and we had to model the outcome to fill in data gaps for those missing values in both countries and years. Moreover, modelled estimates were associated with wide CrIs of countries that had only a single data point, leading to results with low convergence. Third, we examined the national coverage of deworming among pregnant women using nationally representative data that includes all pregnant women in the country, while the WHO recommends deworming specifically for pregnant women residing in intestinal parasitosis endemic areas, defined as regions where school-aged children (SAC) are in need of treatment [13]. Consequently, we possibly underestimated the national coverage in many countries. Fourth, the type of deworming medicine taken by the mother is not specified in the survey data, which raises concern regarding which parasite was targeted in the context of the deworming programme. Since PC coverage is reported by target diseases, and the endemicity is different by species, the outcome variable of this study (i.e. ‘Deworming’) may be complex to interpret. Finally, we established the projections under the assumption of no major change to the existing health care system, and thus could not factor in potential unforeseen impacts of health policies, shifts in donor support, and unforeseen crises such as natural disasters, conflicts, or emergent pandemics. For example, the impact of the coronavirus disease 2019 (COVID-19) pandemic on these projections remains uncertain. LMICs encountered challenges and disruptions to their health care systems due to the pandemic, particularly in maternal health services. Anna et al. [2] utilised mathematical models to forecast the influence of COVID-19 on NTDs control, suggesting that delays might not be significant if appropriate compensatory measures are implemented. Notably, we incorporated the latest GDP per capita projections from the IMF, which might have allowed our model to incorporate the potential effects of COVID-19. Nevertheless, our main objective was to provide insights into each country’s progress towards expanding deworming coverage for pregnant women to inform policymakers, which contributes to addressing the current deficiencies and inequality within the existing programme and health system architecture. Recognising that the pandemic could intensify health inequalities, particularly for pregnant women, is important for gathering additional data on how COVID-19 has affected the implementation and efficacy of deworming programs. Future research should also focus on understanding its impact on the health outcomes of dewormed pregnant women and on the accessibility of deworming services. This will help in developing more resilient health systems and targeted interventions to ensure continued care and support for pregnant women in the face of pandemics and other widespread health emergencies.

Expanding research endeavours is crucial for providing policymakers with evidence-based insights for fortifying deworming programmes and policies. Adopting longitudinal studies or implementing real-time data collection methods would help significantly by countering recall bias and increasing the accuracy of reporting concerning deworming practices during pregnancy. Standardising data collection methodologies across nations contending with endemic intestinal parasitosis is likewise of great importance, as it would ensure consistent and comprehensive deworming coverage data among pregnant women. Emphasis should be placed on harmonising national deworming coverage with the WHO guidelines, especially in countries endemic to intestinal parasites, to ensure adequate coverage for pregnant women requiring these interventions. Investigating the correlation between specific deworming medications and their efficacy against distinct parasitic species necessitates detailed medication data intertwined with prevalence information for these parasites. These avenues of research could not only to address the limitations identified in our study, but also significantly help with improving maternal health services and addressing disparities, particularly during health emergencies.

## CONCLUSIONS

Although substantial progress has been made during the past two decades on deworming coverage among pregnant women in LMICs at the national level, many countries still might fail to achieve the WHO target of 75% national coverage by 2030. Furthermore, large socioeconomic disparities are projected to remain in most countries. We also found determinant factors in the deworming coverage among pregnant women, indicating that maternal services such as ANC can serve as a great opportunity to offer medicine for intestinal parasites. Thus, additional investments in the health sector towards the expansion of deworming programmes, along with integration with existing health services, are urgently required. Moreover, countries that we observed are off-track of the WHO target coverage by 2030 should prioritise expansion of deworming activity and reduction of inequality through the introduction of effective policies and strengthening programmes within the context of ‘Leave No One Behind’ agenda.

## Additional material


Online Supplementary Document
