# Supplementary material for: A comprehensive assessment of deworming coverage among pregnant women in low- and middle-income countries, 2000–30
Source: J Glob Health. 2024 Mar 1;14:04002. doi: 10.7189/jogh.14.04002 (PMC10902802; doi:10.7189/jogh.14.04002)
Supplement: Online Supplementary Document [file jogh-14-04002-s001.pdf]

Supplementary Document

A comprehensive assessment of deworming coverage among pregnant women in low- and middle-income countries, 2000-2030

Miho Sassa, PhD, Daisuke Yoneoka, PhD, Chris Fook Sheng Ng, PhD, Alton Quan Cao, Ganan Devanathan, Masahiro Hashizume, PhD, Shuhei Nomura, PhD

Appendix: The statistical model used

Methods 1: Bayesian hierarchical model for estimating the trend and projection

Bayesian hierarchical linear regression model was applied in the study to fit the models for past trends and develop projections of future deworming coverage among pregnant women at the national level, wealth quintile, place of residence, and maternal education level. First, we created a complete data set of all covariates, including projections up to 2030, since 2000.

The Bayesian models allowed for the estimation of the probability of achieving the WHO deworming target at the national level, while adjusting for covariates. This was achieved by using the posterior distribution in the Bayesian model, which can reflect the probability of the distribution associated with the data (this is often relatively less straightforward in other classical statistical models).[1] A major advantage of using Bayesian methods is the ability to combine existing knowledge of parameters with evidence from data. Moreover, the Bayesian model is favored for analysis of multilevel hierarchical structured models, such as in this study, which enables flexibility in specifying hierarchies of parameters using priors, and the ability to handle small samples.[2,3]

In this study, the trends and projections of outcome variables in countries were nested within each region, which was further nested inside the global level within in the Bayesian model. The global level represents the overall included countries and regions in the model, referring to the categories defined by the United Nations Statistics Division. A model with this hierarchical structure can compensate for limited data for a particular country by borrowing and compensating information from neighboring countries. A similar hierarchical structure has been used in previous DHS studies.[4-6]

Models for covariates preparation

Covariates at the country level, defined x, included in the final Bayesian models were: year, SDI, ratio of government health spending per GDP, ratio of access to safe water, ratio of access to improved sanitation, ratio of skilled health workforce, and GDP per capita. Some covariates had missing years since 2000, and predictions up to 2030 were not available for some of them. Therefore, models were constructed for each covariate to obtain a complete dataset, including predictions through 2030 for those covariates. Since the government health spending per GDP data up to 2030 was available from Global Health Data Exchange (GHDx), we did not make predictions up to 2030 but used the values extracted directly from GHDx. In other words, for these individual models, each covariate was used as the dependent variable, with only the "year" variable used as the explanatory variable. The model equation is as follows:

$$x_{ij} = \alpha_j + \beta_{1,j}\, year_{ij} + \beta_{2,j}\, sdi_{ij} + \epsilon_{ij},$$
$$\epsilon_{ij} \sim N(0, \tau^2),$$

$$\tau^2 \sim Gamma(0.01, 0.01),$$
$$\alpha_j, \beta_{1,j}, \beta_{2,j} \sim N(\mu, \sigma^2),$$

$$\mu \sim N(0, 10000), \qquad \sigma^2 \sim Gamma(0.001, 0.001)$$

where x_{ij} is a covariate of interest (logit transformation, $logit(x) = \log(\frac{x}{1-x})$, was employed if necessary for variables with the support of [0,1]) in year i for j^{th} country, α_{jk} is the random intercept of the jth country in k^{th} region, β_js are the random slope of the jth country and ε_{ijk} is the (Gaussian) error term. sdi_{ij} was predicted until 2030 using the same model without sdi term a priori. A Markov chain Monte Carlo (MCMC) algorithm was used to estimate the posterior distribution of the parameter with two chains. A total of 500 samples were discarded as burn-in and 1000 samples from each chain was obtained from 10,000 iterations with a thinning rate of 10. Inverse transformation was employed to obtain estimated values on the normal scale.

Models for outcome variable

The outcome variable, deworming coverage among pregnant women, was estimated and projected up to 2030 at the national level, as well as across wealth quintiles, place of residence, and level of maternal education using different models. The proportion of the coverage was transformed by the logit transformation. The model was of the following form:

$$y_{jkl} \sim N(\mu_{jkl}, \tau^2)$$
$$\mu_{jk} = \beta_{0,jk} + \beta_{1,jk} year_{jk} + \beta_{2,jk} SDI_{jk} + \beta_{3,jk}\, GHE_{jk} + \beta_{4,jk}\, HW_{jk}$$
$$+ \beta_{5\ jk}\, WAT_{jk} + \beta_{6,jk}\, SAN_{jk} + \beta_{7,jk}\, GDP_{jk} + \epsilon_{jkl}$$
$$\beta_{jk} \sim N(\beta_j, \sigma_j^2)$$
$$\beta_j \sim N(\beta, \sigma^2)$$
$$\beta \sim N(0, 10000)$$
$$\sigma_j, \sigma \sim Gamma(0.001, 0.001)$$
$$\tau^2 \sim Gamma(0.001, 0.001),$$

where *i* = year, *k* = country and *j* = region, GHE is the government health spending per GDP, *HW* is the skilled health workforce, *WAT* is the access to the safe water, *SAN* is the access to adequate sanitation, *and GDP* is GDP per capita.

The same model was applied after stratifying countries by wealth quintile and place of residence separately. The MCMC algorithm with two chains was used for the Bayesian hierarchical models to obtain samples from the posterior distribution of the parameters. A total of 500 samples were discarded as burn-in and 1000 samples from each chain were obtained from 10,000 iterations with a thinning rate of

10. The posterior predicted distributions were used to obtain the back-transformed predicted coverage of outcome variable for each country up to 2030. The 95% credible intervals (CrIs) for all indices were estimated using the 2.5th and 97.5th percentiles of the posterior distribution of the parameter. All models for national-level, across wealth quintile, place of residence, and level of maternal education included the same covariates. Bayesian models were implemented with JAGS 4.13 within R version 4.0.5.

Methods 2: Sensitivity analysis of the Bayesian hierarchical models

Sensitivity analysis for the Bayesian modeling was performed to examine estimated values by altering priors for the hyperparameters.[7] The median absolute differences between the two sets of results and Deviance Information Criterion (DIC) values were compared by excluding country-level predictors to assess the role of country-specific covariates.

The alternative priors are as follows : the half-Cauchy distribution, $HalfCauchy(\kappa, -2)$, $\kappa = z/\sqrt{\chi}$, $\chi \sim Gamma(0.5, 0.5), z \sim HalfNormal(0, 0.001)$ (truncated at 0) was applied instead of gamma distribution in the above model. The median absolute differences between the two sets of results and DIC values were compared after altering prior distribution.

National level estimates of Gelman Rubin Potential scale reduction factors (PSRF) were calculated to check the model's convergence.[8] A PSRF value close to 1 indicates a good convergence of the model.

Methods 3: Determinant analysis

For the determinant analysis on the individual level, the latest DHS survey data were aggregated for each country. A data clearing process was implemented to eliminate individuals with missing values for the variables included. Univariable analysis was conducted applying Bayesian multilevel logistic regression with random intercept country and region (country was nested in regions) to see the association between outcome variable and determinant variables. Multivariable Bayesian multilevel logistic regression with random intercept country and region was applied for the determinant analysis. Multilevel models account for the dependent nature of data across hierarchies, such as individuals nesting within countries and regions. In this study, multilevel models were used to examine whether variables differed by country and region based on the results of multicounty surveys. The standard assumption of the model is that outcome of interest follows a Bernoulli distribution. Similar to the conventional logistic regression model, the outcome p_{ijk} is modeled using the logit link function as follow. The three-level model is given by:

$$y_{ijk} \sim Bernoulli(p_{ijk})$$

$$logit(p_{ijk}) = \beta_{0ijk} + \sum_{h=1}^{H} \beta_h X_{hi}$$

$$\beta_{0ijk} \sim N(\beta_{0jk}, V_{00}), \quad V_{00} \sim InvGamma(0.001, 0.001)$$
$$\beta_{0jk} \sim N(\beta_{0k}, V_0), \quad V_0 \sim InvGamma(0.001, 0.001)$$
$$\beta_{0i} \sim N(\beta_0, U_0), \quad U_0 \sim InvGamma(0.001, 0.001),$$

where $Normal(a, b)$ is the normal distribution with mean a and variance b, $InvGamma(a, b)$ is the inverse gamma distribution with shape a and location b, p_{ijk} is the probability of the binary outcome (deworming during last pregnancy) for the ith pregnant women in country j and region k, and the intercept term β_{0ijk} is assumed to hierarchically follow normal distribution. Uniform prior for regression coefficients for slopes, $\beta_h \sim Uniform(-10, 10)$ for the mean parameter. In the same modeling technique, I conducted the univariable analysis by using one of each X_{hi} in the above equation.

The MCMC algorithm with two chains was used for the models to obtain 1000 samples from the posterior distribution of the parameters. A total of 2,500 samples were discarded as burn-in, and 1,000 samples from each chain were obtained from 3,500 iterations with a thinning rate of 10.

Correlation analysis was carried out to investigate the potential relationships between the variables. Pearson's correlation coefficient was employed to quantify the linear association between the variables. The correlation coefficient ranges from -1 to 1, with 0 indicating no linear correlation. In this study, absolute correlation coefficient above 0.7 considered as high correlation.[9] Determinant analysis was performed in Stata MP version 17.0.

Reference

1. Gelman A, Carlin, J.B., Stern, H.S., Dunson, D.B., Vehtari, A., & Rubin, D.B. Bayesian Data Analysis (3rd ed.). 3rd Edition ed. New York, United States: Chapman and Hall; 2013.
2. Danaei G, Finucane MM, Lin JK, et al. National, regional, and global trends in systolic blood pressure since 1980: systematic analysis of health examination surveys and epidemiological studies with 786 country-years and 5.4 million participants. *Lancet* 2011; **377**(9765): 568-77.
3. Ntzoufras I. Baysesian modeling using WinBUGS. New Jersey, United States; 2011.

4. Ganatra B, Gerdts C, Rossier C, et al. Global, regional, and subregional classification of abortions by safety, 2010-14: estimates from a Bayesian hierarchical model. *Lancet* 2017; **390**(10110): 2372-81.

5. Alkema L, Kantorova V, Menozzi C, Biddlecom A. National, regional, and global rates and trends in contraceptive prevalence and unmet need for family planning between 1990 and 2015: a systematic and comprehensive analysis. *Lancet* 2013; **381**(9878): 1642-52.

6. Bearak J, Popinchalk A, Alkema L, Sedgh G. Global, regional, and subregional trends in unintended pregnancy and its outcomes from 1990 to 2014: estimates from a Bayesian hierarchical model. *Lancet Glob Health* 2018; **6**(4): e380-e9.

7. Gupta N, Kiran U, Bhal K. Teenage pregnancies: obstetric characteristics and outcome. *Eur J Obstet Gynecol Reprod Biol* 2008; **137**(2): 165-71.

8. Harrington SM, Wishingrad V, Thomson RC. Properties of Markov Chain Monte Carlo Performance across Many Empirical Alignments. *Mol Biol Evol* 2021; **38**(4): 1627-40.

9. Schober P, Boer C, Schwarte LA. Correlation Coefficients: Appropriate Use and Interpretation. *Anesth Analg* 2018; **126**(5): 1763-8.

Table S1: Study countries and observed national deworming coverage in pregnant women

Country	Survey year	Sample size	Deworming coverage (%)		
			National	Urban	Rural
**East, South and South-East Asia**					
Afghanistan	2015	19,666	3.0	2.9	3.1
Cambodia	2014	5,899	72.2	62.0	73.9
	2010	6,448	44.5	38.9	45.6
	2005	6,142	10.7	14.3	10.1
India	2016	190,797	18.0	21.4	16.6
	2006	36,850	3.8	4.4	3.5
Maldives	2009	3,255	14.6	6.8	18.0
Myanmar	2016	3,867	55.3	59.8	54.0
Nepal	2016	4,006	69.2	67.9	70.9
	2011	4,079	55.1	49.8	55.7
	2006	4,181	20.3	16.5	20.9
Pakistan	2018	8,286	1.8	1.5	2.0
	2013	7,461	2.5	2.9	2.3
Philippines	2017	7,992	4.1	2.9	5.0
	2013	5,301	4.7	4.2	5.2
	2008	4,712	3.8	2.1	5.5
Timor-Leste	2016	4,916	16.0	16.2	15.9
	2010	5,999	13.3	6.7	15.4
**Oceania**					
Papua New Guinea	2018	6,543	18.1	18.1	18.1
**Europe, Central and Western Asia**					
Albania	2018	2,356	1.9	1.8	1.9
	2009	1,341	2.5	2.3	2.5
Armenia	2010	1,163	0.6	1.0	0.0
Azerbaijan	2006	1,698	3.1	2.8	3.4
Kyrgyzstan	2012	3,123	7.8	11.6	6.1
Tajikistan	2017	4,238	1.7	2.2	1.5
Ukraine	2007	1,097	9.3	11.0	5.8
**Latin, Central, and South America and the Caribbean**					
Dominican Republic	2013	2,960	10.6	10.0	12.2
Guatemala	2015	9,524	6.3	6.1	6.4
Guyana	2009	1,583	17.2	18.5	16.8
Haiti	2017	5,005	9.8	10.3	9.5
	2012	5,414	14.0	14.7	13.6
	2006	4,237	6.9	9.0	5.7
Honduras	2012	8,715	6.0	5.1	6.8

	2006	8,082	6.9	7.0	6.8
Peru	2012	8,000	2.8	2.1	4.0
	2011	7,561	2.6	2.2	3.6
	2010	7,665	2.9	2.2	4.4
	2009	8,446	2.5	2.2	3.1
	2008	9,716	3.4	2.6	4.6
	2006	9,716	0.8	0.7	1.0
**Middle East and North Africa**					
Egypt	2014	11,491	3.3	3.1	3.4
Yemen	2013	10,459	3.4	2.2	4.0
**Sub-Saharan Africa**					
Angola	2016	8,947	49.2	62.0	26.3
Benin	2018	8,994	64.6	72.6	59.4
	2012	9,111	74.6	81.3	70.0
Burkina Faso	2010	10,364	24.3	26.2	23.8
Burundi	2017	8,660	66.4	71.5	65.8
	2010	4,916	30.9	29.8	31.0
Cameroon	2018	6,463	31.1	37.2	25.6
	2011	7,610	37.2	43.1	32.3
Chad	2015	11,048	23.3	32.9	20.9
Comoros	2012	2,016	62.4	65.1	61.3
Congo	2012	6,463	85.8	91.4	76.3
DRC	2014	11,271	55.9	68.5	50.1
Ethiopia	2016	7,193	5.7	7.7	5.4
	2011	7,764	5.5	8.5	5.0
Gabon	2012	4,143	70.7	73.7	52.8
The Gambia	2020	5,799	40.6	37.0	47.9
	2013	5,374	40.3	36.0	44.6
Ghana	2014	4,294	39.4	36.2	42.2
	2008	2,147	34.9	34.4	35.2
Guinea	2018	5,530	38.7	51.6	33.2
	2012	4,980	29.4	49.2	21.6
Kenya	2014	7,164	31.3	31.6	31.1
	2009	4,082	17.0	19.6	16.4
Liberia	2020	4,267	63.8	65.3	61.8
	2013	5,348	57.9	59.8	55.7
	2007	3,996	28.5	34.9	25.3
Madagascar	2009	8,569	39.4	40.3	39.3
Malawi	2016	13,448	51.6	48.4	52.1
	2010	13,776	27.4	29.9	26.9

Mali	2018	6,368	49.9	46.1	51.0
	2013	6,723	27.2	33.2	25.6
Mozambique	2011	7,623	28.3	41.7	22.7
Namibia	2013	3,962	6.9	6.1	7.6
	2007	4,005	7.4	7.0	7.7
Niger	2012	7,680	51.5	57.3	50.5
Nigeria	2018	21,792	16.7	18.6	15.5
	2013	20,192	14.4	19.3	11.7
	2008	17,931	9.6	11.7	8.7
Rwanda	2020	6,167	43.0	41.4	43.3
	2015	5,952	49.3	51.3	48.9
	2010	6,328	39.1	35.5	39.6
	2008	3,568	17.9	20.1	17.5
STP	2009	1,432	52.0	54.7	49.3
Senegal	2019	4,345	55.4	63.8	50.0
	2018	4,703	44.8	51.0	40.6
	2017	8,486	40.1	37.7	41.8
	2016	9,254	33.1	38.2	29.7
	2015	4,679	31.3	36.4	28.2
	2014	4,484	27.0	24.2	29.2
	2013	4,470	25.2	30.1	22.3
	2011	8,147	25.0	26.8	23.7
Sierra Leone	2019	7,377	83.5	86.9	81.4
	2013	8,524	72.4	75.9	71.1
	2008	3,980	43.8	45.7	43.1
Tanzania	2016	7,049	63.1	73.4	58.6
Togo	2014	5,006	57.2	64.4	53.0
Uganda	2016	10,263	59.9	62.2	59.2
	2011	4,909	49.9	53.7	49.2
	2006	5,004	26.8	27.3	26.8
Zambia	2018	7,372	77.4	81.7	74.6
	2014	9,347	64.4	69.9	61.0
	2007	4,148	36.0	50.4	29.1
Zimbabwe	2015	4,833	3.4	2.2	3.9
	2011	4,397	2.3	0.9	2.9
**Total**		**924,277**			

*DRC: Democratic Republic of the Congo, STP: Sao Tome and Principe

Table S2: Coverage of deworming among pregnant women according to level of education, 2000-2030

Country	Estimate and predicted coverage (95% credible intervals)							
	2000		2010		2020		2030	
	Urban	Rural	Urban	Rural	Urban	Rural	Urban	Rural
Afghanistan	2.4 (0.0-22.8)	2.3 (0.0-21.6)	2.8 (0.6-8.4)	2.8 (0.6-8.2)	3.8 (0.9-9.8)	3.8 (0.9-9.8)	10.2 (0.2-51.2)	10.1 (0.2-53.0)
Albania	2.3 (0.6-8.1)	2.3 (0.6-8.3)	2.4 (1.8-3.2)	2.4 (1.7-3.2)	1.8 (1.3-2.4)	1.7 (1.2-2.4)	1.3 (0.6-2.4)	1.3 (0.6-2.3)
Angola	28.9 (0.6-88.6)	14.5 (0.2-69.3)	55.4 (19.9-85.6)	28.4 (7.0-63.0)	43.2 (17.9-70.3)	18.5 (5.3-38.8)	57.1 (6.9-95.9)	34.3 (2.1-87.8)
Armenia	0.3 (0.0-2.0)	0.0 (0.0-0.0)	0.8 (0.6-1.0)	0.0 (0.0-0.0)	3.0 (0.3-12.9)	0.0 (0.0-0.0)	14.8 (0.1-78.4)	0.1 (0.0-0.6)
Azerbaijan	1.2 (0.1-4.8)	1.3 (0.1-5.3)	8.9 (2.3-22.4)	9.6 (2.4-25.0)	12.5 (0.6-50.0)	13.3 (0.7-54.8)	25.7 (0.2-93.3)	26.6 (0.2-94.1)
Benin	83.7 (61.9-95.4)	76.0 (52.1-92.8)	81.1 (75.1-86.2)	72.1 (63.7-79.8)	64.8 (49.8-77.0)	52.8 (37.7-69.0)	45.3 (15.4-76.3)	34.3 (10.1-66.5)
Burkina Faso	10.6 (0.7-39.5)	9.5 (0.5-38)	26.8 (20.9-33.2)	24.1 (17.8-32.1)	46.9 (9.0-89.7)	44.0 (7.4-88.5)	58.5 (3.0-99.2)	56.4 (2.7-99)
Burundi	3.8 (1.2-8.2)	3.4 (1.1-7.5)	33.0 (26.7-39)	30.5 (24.0-38.0)	77.1 (68.7-84)	74.9 (64.7-83)	96.3 (92.3-98.5)	95.9 (91.7-98.3)
Cambodia	3.4 (1.2-8.2)	3.6 (1.3-8.5)	41.5 (36.5-46.6)	43.2 (37.4-49.3)	90.6 (84-94.9)	91.1 (85.1-95.2)	98.1 (93.2-99.7)	98.3 (93.7-99.7)
Cameroon	33.4 (10.6-64.3)	24.9 (7.7-53.3)	41.1 (33.5-49.3)	30.8 (23.8-38.8)	32.1 (20.8-43.5)	23.3 (14.4-33.4)	31.0 (14.0-53.2)	22.6 (9.1-41.7)
Chad	16.2 (0.0-91.0)	13.0 (0.0-86.2)	25.2 (6.9-49.9)	18.0 (4.2-40.2)	35.6 (14-68.2)	26.4 (9.4-55.9)	52.4 (4.9-97.8)	44.6 (3.1-96.4)
Comoros	32.2 (2.3-85.4)	29.7 (2.2-84.1)	61.6 (50.0-72.4)	57.7 (45.0-70.3)	66.4 (32.5-90.2)	63.1 (26.8-89.2)	66.7 (8.5-98.6)	64.2 (6.9-98.6)
Congo	51.0 (4.7-95.6)	34.4 (1.8-89.4)	86.1 (75.6-93.4)	70.5 (53.2-83.9)	84.4 (52.7-97.6)	69.6 (30.1-94.1)	86.9 (27.6-99.8)	77.3 (13.2-99.6)
DRC	64.2 (6.8-98.9)	54.9 (3.7-98.1)	62.7 (35.6-85.9)	49.2 (22.2-76.1)	70.0 (40.8-89.4)	57.3 (28.7-82.4)	72.7 (14.9-99.0)	63.4 (8.8-98.4)
Dominican Republic	8.1 (0.2-46.7)	8.9 (0.2-51.1)	9.2 (4.6-17.1)	10.4 (4.7-19.4)	18.0 (3.6-46.0)	19.8 (4.1-48.1)	38.1 (1.0-95.0)	40.1 (1.1-95.4)
Egypt	3.0 (0.1-16.8)	3.1 (0.1-17.4)	2.8 (1.1-5.7)	2.8 (1.1-5.9)	5.0 (1.3-12.9)	5.1 (1.4-13.0)	14.6 (0.3-65.8)	14.8 (0.3-64)
Ethiopia	6.1 (0.0-48.4)	4.7 (0.0-41.2)	6.2 (3.4-9.5)	4.3 (2.4-7.0)	7.6 (3.7-13.6)	5.4 (2.5-10.0)	9.9 (0.6-41.5)	7.3 (0.4-32.1)
Gabon	46.9 (4.7-93.2)	31.8 (2.1-84.6)	69.5 (56.7-80.4)	49.4 (34.9-64.2)	69.4 (35.6-92.2)	51.2 (17.5-83.6)	73.9 (14.1-99.4)	61.0 (6.8-98.6)
Ghana	21.9 (2.6-64.5)	23.2 (2.6-65.9)	34.7 (24.1-48.0)	36.6 (24.7-50.2)	43.2 (28.1-57.7)	45.3 (30.0-60.6)	43.8 (19.1-69.5)	45.8 (20.4-72.2)
Guatemala	1.9 (0.0-12.3)	1.9 (0.0-13.3)	3.4 (1.1-8.1)	3.4 (1.0-8.8)	11.8 (4.1-26.4)	11.7 (4.0-26.5)	34.1 (2.2-89.2)	33.8 (2.3-89.5)
Guinea	29.6 (9.4-63.5)	15.9 (4.3-42.9)	41.2 (30.0-52.9)	23 (14.5-32.9)	55.4 (46.1-65.3)	34.5 (25.9-44.3)	67.1 (42.7-86.4)	47.5 (22.8-72.9)
Guyana	13.6 (0.7-54.8)	12.3 (0.6-50.5)	20.6 (13.9-28.1)	18.4 (11.6-26.2)	38.0 (2.9-90.7)	35.6 (2.6-89.7)	60.9 (0.8-99.8)	59.1 (0.7-99.7)
Haiti	15.5 (6.2-28.8)	13.0 (4.8-24.7)	17.7 (9.6-27.7)	14.8 (7.9-23.9)	10.1 (6.6-14.2)	8.3 (5.6-12.2)	14.4 (7.0-23.9)	12.0 (5.8-20.8)
Honduras	8.5 (4.7-14.1)	9.1 (5.1-15.2)	5.9 (4.2-7.7)	6.4 (4.6-8.6)	4.5 (2.0-8.7)	4.9 (2.2-9.8)	3.7 (0.8-10.6)	4.0 (0.8-12.0)
India	1.4 (0.6-2.8)	1.1 (0.5-2.3)	10.5 (7.1-14.3)	8.3 (5.4-11.8)	29.8 (20.3-39.6)	24.8 (16.7-34.2)	65.4 (44.9-81.0)	59.7 (40.5-76.9)

Kenya	5.8 (2.5-11.1)	5.2 (2.1-10.6)	21.0 (16.5-25.5)	18.9 (14.5-23.3)	50.3 (35.2-65.2)	47.1 (31.9-62)	70.7 (39.8-91.2)	68.1 (36.5-90.6)
Kyrgyzstan	3.4 (0.0-22.1)	2.1 (0.0-13.7)	7.4 (3.4-13.3)	4.3 (2.1-8.0)	18.3 (3.6-48.5)	11.8 (2.0-37.0)	39.4 (1.6-94.9)	30.5 (1.0-92.1)
Liberia	17.6 (12.1-24.3)	14.3 (9.5-20.3)	44.4 (32.9-56.1)	38.4 (27.5-49.5)	66.7 (59.9-72.9)	61.0 (53.1-67.9)	84.9 (71.9-93.4)	81.5 (66.7-91.6)
Madagascar	33.9 (4.9-78.3)	32.1 (4.6-77.2)	41.2 (30.2-51.8)	38.9 (27.6-51.3)	42.0 (3.0-92.4)	40.3 (2.7-92.1)	46.9 (0.5-98.9)	45.6 (0.5-98.9)
Malawi	10.4 (0.9-43.9)	10.2 (0.9-42.7)	28.9 (23.6-35.4)	28.3 (21.9-35.5)	67.7 (47.8-82.8)	67 (45.7-82.7)	82.1 (39.1-98.5)	81.7 (36.7-98.5)
Maldives	7.0 (0.3-32.8)	12.7 (0.6-50.9)	10.0 (6.5-14.9)	19.4 (12.5-28.4)	17.9 (1.8-60.3)	29.5 (3.5-78.4)	36.0 (0.5-96.7)	47.1 (1.1-98.5)
Mali	12.4 (0.4-76.6)	11.7 (0.4-72.2)	23.4 (14.1-36.1)	21.6 (12.6-35.0)	53.5 (41.6-64.8)	50.9 (39.4-62.6)	71.0 (24.9-94.5)	69.2 (24.5-94.1)
Mozambique	32.2 (0.3-98.1)	23.9 (0.1-96.7)	33.2 (23.0-44.5)	19.6 (12.3-29.1)	35.8 (5.8-80.1)	23.5 (3.0-65.1)	46.6 (2.2-97.8)	35.5 (1.1-95.0)
Myanmar	41.2 (0.4-98.6)	38.3 (0.3-98.3)	73.5 (4.4-99.9)	70.8 (3.6-99.8)	62.6 (38.9-82.3)	57.5 (32.4-80.9)	61.5 (10.3-97.4)	57.6 (6.9-97.0)
Namibia	5.3 (2.1-12.9)	6.0 (2.5-14.4)	7.5 (5.1-9.8)	8.5 (5.8-11.5)	4.5 (1.7-12.0)	5.1 (1.9-13.5)	3.6 (0.5-14.6)	4.1 (0.6-15.3)
Nepal	6.2 (3.9-9.2)	7.3 (4.6-10.9)	42.2 (36.5-48.1)	46.4 (40.1-53.0)	79.6 (69.5-87.8)	82.1 (71.9-89.2)	95.6 (90.5-98.2)	96.2 (92-98.5)
Niger	42.4 (4.0-88.6)	37.8 (3.3-86.8)	55.2 (41.0-68.5)	49.1 (33.9-64.5)	54.3 (19-87.6)	48.9 (15.8-85.8)	52.8 (3.2-98.0)	48.7 (2.5-97.5)
Nigeria	2.9 (0.4-14.0)	2.1 (0.3-11.1)	13.1 (10.6-16.2)	9.6 (7.4-12.1)	21.3 (16.1-27.1)	16 (11.8-21.2)	45.8 (18-76.9)	37.9 (13.3-70.5)
Pakistan	5.9 (0.4-26.0)	6.0 (0.4-26.0)	2.8 (1.7-4.5)	2.9 (1.7-4.5)	1.4 (0.8-2.4)	1.4 (0.8-2.4)	0.8 (0.2-2.1)	0.8 (0.2-2.2)
Papua New Guinea	12.2 (0.0-79.9)	11.9 (0.0-81.0)	13.6 (1.7-47.8)	13.3 (1.7-45.4)	19.9 (12-30.1)	19.4 (11.6-29)	35.9 (3.1-86.2)	35.3 (2.8-85.6)
Peru	0.3 (0.1-0.5)	0.4 (0.1-0.8)	2.0 (1.7-2.5)	3.3 (2.8-3.9)	3.1 (0.9-7.9)	5.0 (1.4-11.7)	7.6 (1.1-24.7)	11.6 (1.9-33.6)
Philippines	1.7 (0.8-3.1)	2.9 (1.3-5.3)	2.8 (2.1-3.6)	4.6 (3.4-5.9)	3.2 (2.1-4.6)	5.3 (3.4-7.7)	3.4 (1.4-6.7)	5.6 (2.3-11.1)
Rwanda	3.6 (0.5-10.8)	3.5 (0.5-10.8)	34.1 (28.3-39.6)	33.8 (27.8-39.6)	48.2 (41.9-54.7)	47.8 (41.2-54.8)	65.7 (52.9-76.6)	65.3 (52.7-76.5)
STP	41 (5.1-88.5)	37.1 (4.2-85.7)	58.0 (45.4-69.4)	52.9 (39.6-65.8)	75.9 (22.1-98.2)	72.8 (18.7-97.9)	77.5 (5.6-99.9)	75.4 (5.1-99.8)
Senegal	2.3 (0.9-4.7)	1.8 (0.7-3.9)	20 (16.6-23.5)	16.5 (13.6-19.6)	57.6 (52.2-62.8)	51.8 (46.3-56.8)	89.5 (81.8-94.7)	87.1 (78.1-93.1)
Sierra Leone	27.1 (2-81.9)	23.5 (1.6-75.8)	55.5 (48.6-61.8)	49.2 (42.4-56.0)	86.8 (82.9-90.1)	83.6 (79.0-87.7)	95 (87.9-98.4)	93.6 (84.4-98.0)
Tajikistan	1.0 (0.0-7.8)	0.8 (0.0-5.8)	1.4 (0.2-5.3)	1.0 (0.1-3.6)	2.9 (1.3-5.8)	2.1 (0.9-4.2)	13.1 (0.5-56.3)	10.3 (0.4-47.1)
Tanzania	44.0 (2.1-95.0)	35.2 (1.3-92.2)	61.7 (31.3-85.8)	49.1 (21.4-78.0)	75.2 (56.3-88.6)	64.0 (41.7-82.4)	78.5 (23.3-98.9)	70.5 (15.8-98.3)
The Gambia	31.3 (4.1-75.9)	37.7 (6.0-80.4)	40.7 (29.2-53.1)	49.1 (36.3-61.5)	38.5 (31.9-45.5)	46.9 (39.1-55.0)	50.6 (19.4-83.0)	58.2 (24.2-87.5)
Timor-Leste	5.2 (0.1-27.2)	7.0 (0.2-36.3)	9.4 (6.9-12.2)	13.1 (9.7-16.8)	18.1 (6.9-38.0)	24.0 (9.5-48.7)	27.2 (2.1-83.8)	33.0 (2.9-89.4)
Togo	44.3 (1.5-97.2)	38 (0.8-95.5)	56.8 (34.1-74.9)	46.8 (25.6-67.3)	67.1 (35.4-90.0)	58.1 (26.6-86.3)	70.2 (12.0-99.0)	63.6 (9.1-98.6)
Uganda	18.7 (5.5-53.5)	17.3 (4.9-50.8)	47.7 (41.6-54.1)	45.2 (38.3-51.6)	70.0 (59.3-79.4)	67.8 (57.4-77.5)	81.3 (48.3-96.9)	79.9 (46-96.5)
Ukraine	3.9 (0.2-20.1)	2.2 (0.1-12.5)	12.0 (6.2-21.0)	6.8 (3.3-12.6)	23.8 (1.6-74.3)	15.9 (0.8-60.9)	35.4 (0.3-97.4)	27.7 (0.2-95.1)
Yemen	1.8 (0.0-12.3)	2.5 (0.0-19.0)	1.8 (0.7-3.7)	2.8 (1.1-5.9)	3.9 (0.2-19.6)	5.9 (0.2-27.6)	16.4 (0.1-80.2)	21.1 (0.2-87.2)

Zambia	31.9 (1.5-95.5)	24.9 (0.8-92.2)	51.3 (20.7-75.2)	38.9 (12.9-63.6)	80 (70.3-88.8)	70.1 (57.4-82.5)	92.2 (77.2-98.2)	87.6 (64.8-97.0)
Zimbabwe	0.3 (0.1-1.0)	0.7 (0.1-2.1)	0.8 (0.5-1.3)	1.7 (1.0-2.8)	3.7 (1.8-6.8)	7.8 (3.8-13.8)	21.0 (3.2-55.5)	34.4 (6.6-74.1)

DRC: Democratic Republic of the Congo, STP: Sao Tome and Principe

Table S3: Coverage of deworming among pregnant women by wealth quintile, 2000-2030

	Estimated and predicted coverage (95% credible intervals)							
	2000		2010		2020		2030	
Country	Poorest	Richest	Poorest	Richest	Poorest	Richest	Poorest	Richest
Afghanistan	0.6 (0.0-4.4)	1.3 (0.0-9.1)	1.5 (0.3-3.9)	3.4 (0.8-8.4)	1.6 (0.4-4.5)	3.7 (1.1-9.6)	4.4 (0.1-31.9)	8.3 (0.2-49.8)
Albania	1.6 (0.3-4.4)	1.1 (0.3-3.3)	2.2 (1.6-3.0)	1.6 (1.2-2.2)	1.8 (1.2-2.6)	1.3 (0.9-1.9)	1.6 (0.8-2.8)	1.1 (0.6-1.9)
Angola	11.1 (0.1-64.2)	36.0 (0.8-94.0)	24.5 (4.9-62.5)	69.2 (32.8-93.8)	11.9 (3.5-29.3)	52.4 (25.4-79.5)	27.3 (1.3-81.4)	65.7 (11.3-97.7)
Armenia	0.0 (0.0-0.0)	0.4 (0.0-2.5)	0.0 (0.0-0.0)	0.9 (0.6-1.3)	0.0 (0.0-0.0)	3.6 (0.3-15.6)	0.1 (0.0-1.1)	17 (0.1-86.6)
Azerbaijan	0.5 (0.0-2.2)	0.9 (0.1-4.0)	7.9 (1.8-23.6)	13.5 (3.2-33.3)	12.1 (0.8-49.3)	19. (1.5-64.2)	29.3 (0.3-94.3)	38.1 (0.8-96.6)
Benin	60.3 (29.6-84.3)	90.8 (76.5-97.7)	56.6 (45.8-66.5)	90.4 (86.4-93.4)	36.6 (23.4-52.1)	80.2 (68.7-88.6)	22.0 (6.2-49.9)	63.1 (31.1-87.7)
Burkina Faso	8.4 (0.5-38.9)	10.2 (0.6-44.5)	22.3 (16.0-29.7)	27.0 (20.3-34.3)	39.6 (4.3-84.7)	44.5 (5.6-88.6)	49.6 (1.4-98.0)	53.6 (1.6-98.5)
Burundi	3.1 (1.2-6.9)	4.2 (1.6-9.0)	28.3 (21.8-35.3)	34.6 (28.3-41.5)	70.6 (60.7-79.1)	76.3 (68.7-83.2)	94.9 (89.9-97.6)	96.2 (92.7-98.2)
Cambodia	2.5 (0.8-6.5)	3.7 (1.2-9.0)	36.1 (29.8-42.2)	46.3 (40.4-52.6)	90.6 (84.4-95.0)	93.6 (89.3-96.5)	98.4 (95.1-99.7)	99 (96.6-99.8)
Cameroon	10.5 (2.3-27.9)	33 (9.6-64.3)	16.4 (11.9-21.8)	47.4 (39.0-56.4)	11.0 (6.2-17.7)	35.9 (24.7-49.6)	12.5 (4.4-26.8)	38.3 (17.2-61.9)
Chad	12.2 (0.0-75.9)	17.5 (0.1-86.0)	16.4 (4.9-35.8)	27.6 (10.1-50.8)	19.3 (6.5-40.5)	31.8 (12.8-58.3)	33.4 (1.8-88.7)	45.4 (3.9-93.7)
Comoros	34.5 (2.6-89.1)	40.9 (3.8-92.5)	58.7 (44.1-72.2)	67.3 (55.9-77.8)	59.7 (24.8-88.4)	67.4 (31.5-91.6)	58.6 (5.2-97.9)	64.5 (7.1-98.6)
Congo	30.8 (1.0-89.4)	58.8 (5.4-97.9)	64.8 (42.5-82.5)	90.9 (81.5-96.4)	61.3 (23.4-91.8)	87.8 (60.6-98.5)	70.4 (10.3-99.4)	88.7 (38.0-99.9)
DRC	48.8 (2.1-98.1)	65 (6.2-99.3)	41.1 (14.8-73.1)	64.9 (34.7-88.6)	52.0 (23.7-79.4)	74.6 (48.4-92.0)	61.2 (8.4-98.0)	77.1 (18.2-99.4)
Dominican Republic	12.1 (0.3-58.4)	7.8 (0.1-44.5)	12.9 (6.1-23.6)	7.3 (3.4-14.1)	22.1 (4.7-52.7)	13.6 (2.5-37.3)	41.6 (1.4-95.3)	32.0 (0.7-92.3)
Egypt	2.8 (0.1-18.4)	2.9 (0.1-17.6)	2.6 (1.0-5.6)	2.6 (1.1-5.8)	4.9 (1.2-12.1)	4.9 (1.3-12.3)	14.6 (0.3-60.5)	14.7 (0.3-62.9)
Ethiopia	2.5 (0.0-24.4)	4.6 (0.0-42.6)	2.7 (1.4-4.6)	5.9 (3.1-10.1)	4.0 (1.9-6.9)	8.5 (4.3-14.9)	6.3 (0.4-28.9)	12.1 (0.8-47.0)
Gabon	28.5 (1.3-80.3)	45.8 (4.3-92.0)	49.9 (32.8-65.1)	73.1 (59.6-83.7)	49.7 (18.2-81.2)	71.1 (37.9-92.8)	61.4 (7.2-98.3)	76.5 (18.1-99.4)
Ghana	12.6 (0.6-52.8)	10.4 (0.4-45.2)	39.1 (23.5-56.9)	33.3 (19.8-49.4)	39.1 (23.6-55.1)	33.3 (19.8-50.1)	40.0 (18.0-65.3)	34.3 (14.5-58.7)
Guatemala	3.3 (0.1-20.9)	2.6 (0.0-16.2)	4.5 (1.4-11.8)	3.3 (1.0-8.6)	11.7 (4.0-25.1)	8.8 (3.0-19.6)	29.1 (1.6-85.7)	24.3 (1.2-79.3)
Guinea	10.0 (3.3-20.2)	29.3 (12.3-50.5)	16.5 (9.3-25.0)	43.1 (30.2-57.2)	28.9 (21.2-37.7)	61.2 (51.8-70.5)	40.4 (17.4-66.7)	70.9 (46.6-89.6)
Guyana	16.3 (0.5-66.7)	11.3 (0.3-53.3)	25.4 (16.6-36.2)	16.6 (10.6-23.8)	46.1 (3.3-93.0)	36.8 (2.0-89.2)	69.7 (1.4-99.9)	64.1 (0.8-99.9)
Haiti	13.1 (5.5-26.3)	19.2 (9.0-35.1)	14.4 (8.0-23.9)	21.1 (12.8-33.7)	6.7 (4.5-9.8)	10.3 (7.0-14.3)	10.7 (5.2-19.0)	16.0 (8.2-27.3)
Honduras	11.1 (6.4-17.9)	8.5 (4.6-14.2)	7.0 (4.8-9.8)	5.3 (3.6-7.1)	5.0 (2.4-9.2)	3.8 (1.7-7.0)	3.6 (0.9-9.2)	2.7 (0.7-7.1)
India	0.9 (0.4-1.9)	1.9 (0.8-3.9)	6.9 (4.4-10.7)	13.4 (8.9-19.2)	19.1 (12.2-27.2)	32.9 (23.1-43.0)	50.2 (34.1-67.4)	67.5 (52.4-80.6)

Kenya	3.4 (1.6-6.4)	5.1 (2.4-9.8)	14.7 (10.7-19.3)	20.7 (16.0-25.5)	41.2 (27.1-55.9)	51.5 (37.4-65.6)	63.7 (31.8-87.7)	72.1 (41.9-91.7)
Kyrgyzstan	2.6 (0.0-20.5)	4.2 (0-30.8)	4.7 (2.1-9.4)	8.2 (3.8-15.5)	14.1 (2.3-39.0)	22.3 (4.3-54.9)	33.5 (1.1-93.6)	43.2 (1.8-96.4)
Liberia	12.2 (8.4-16.8)	18.5 (13.1-24.7)	36.1 (25.3-49)	47.9 (35.4-60.3)	58.2 (51.0-65.1)	69.5 (63.2-74.9)	79.4 (60.2-92.1)	86.1 (70.5-94.8)
Madagascar	28.7 (2.6-79.3)	32.6 (3.2-83.3)	36.5 (24.5-50.7)	41.8 (28.0-56.1)	41.9 (1.7-96.3)	45.7 (2.1-97.3)	48.1 (0.3-99.7)	51.1 (0.5-99.7)
Malawi	12.5 (1.2-41.9)	13.0 (1.4-43.3)	27.3 (21.0-33.7)	28.0 (21.9-34.7)	64.8 (45.1-80.9)	65.7 (46.2-81.6)	73.1 (29.4-97.4)	73.8 (28.9-97.4)
Maldives	7.8 (0.3-39.5)	2.0 (0.1-9.7)	23.4 (14.8-33.3)	5.8 (3.4-8.9)	37.0 (3.7-83.8)	13.4 (0.9-48)	55.4 (1.3-98.8)	32.8 (0.3-94.4)
Mali	12.0 (0.2-60.7)	17.0 (0.4-72.4)	17.8 (9.4-28.5)	26.9 (15.9-39.9)	44.0 (33.2-55.8)	57.3 (46.1-68.3)	56.1 (15.6-91.2)	66.7 (22.3-94.7)
Mozambique	25.2 (0.0-91.3)	44.8 (0.2-97.9)	12.7 (7.6-18.9)	38.5 (26.7-50.4)	21.2 (2.4-64.8)	47.2 (10.8-88.6)	32.9 (1.2-92.0)	55.9 (5.1-98.2)
Myanmar	44.1 (0.5-99.3)	46.3 (0.5-99.4)	79.8 (10.5-100.0)	81.4 (11.9-100.0)	53.6 (26.1-77.2)	57.6 (32.1-79.4)	52.6 (6.2-94.7)	55.8 (7.1-96.0)
Namibia	7.7 (3.5-14.8)	4.3 (1.9-8.4)	12.3 (9.0-16.5)	6.9 (5.0-9.2)	5.5 (2.3-11.5)	3.0 (1.2-6.2)	3.6 (0.8-10.2)	2.0 (0.4-5.6)
Nepal	6.1 (4.2-8.8)	7.1 (4.8-10.2)	40.5 (34.2-47)	44.5 (38.3-50.5)	74.7 (64.3-83.7)	77.7 (67.2-85.7)	94.4 (90.1-97.3)	95.2 (91.3-97.8)
Niger	22.9 (1.7-69.4)	34.6 (3.3-83.7)	37.6 (23.6-53.2)	55.6 (40.5-69.5)	43.1 (12.7-80.9)	59.4 (24.4-89.7)	47.9 (3.1-96.8)	60.2 (6.3-98.6)
Nigeria	0.8 (0.0-4.2)	2.2 (0.1-10.7)	5.6 (4.2-7.3)	14.9 (11.8-18.6)	10.4 (7.7-13.7)	25.5 (19.4-31.4)	33.0 (9.3-64.3)	56.9 (23.6-83.9)
Pakistan	4.4 (0.2-18.6)	7.0 (0.4-29.3)	1.8 (1.1-2.8)	3.1 (2.0-4.8)	1.0 (0.5-1.7)	1.7 (1.0-2.9)	0.6 (0.1-1.5)	1.0 (0.3-2.5)
Papua New Guinea	5.4 (0.0-35.9)	9.3 (0.1-58.1)	7.0 (0.9-21.6)	13.1 (2.0-37.1)	11.9 (6.9-18.9)	21.7 (13.3-32.2)	26.2 (3.0-79.3)	38.5 (5.7-87.6)
Peru	0.4 (0.1-0.8)	0.1 (0.1-0.3)	3.6 (2.9-4.2)	1.3 (1.1-1.6)	2.5 (0.5-6.3)	0.9 (0.2-2.4)	4.2 (0.3-16.0)	1.6 (0.1-6.7)
Philippines	3.5 (1.6-6.7)	1.2 (0.6-2.3)	5.8 (4.3-7.9)	2.1 (1.6-2.8)	6.4 (4.3-9.4)	2.3 (1.6-3.3)	6.3 (2.9-12.3)	2.3 (1.0-4.4)
Rwanda	1.3 (0.3-3.9)	1.5 (0.3-4.6)	34.4 (28.4-40.6)	37.7 (31.9-43.6)	45.3 (38.6-52.4)	48.9 (42.6-55.2)	62.5 (47.7-74.3)	65.7 (51.2-76.0)
STP	41.8 (3.8-88.9)	48.8 (5.4-90.6)	51.0 (37.7-64.0)	59.9 (47.5-70.4)	69.6 (20.8-96.4)	75.5 (24.3-97.4)	71.0 (4.9-99.6)	75.4 (7.2-99.7)
Senegal	1.2 (0.5-2.3)	2.1 (0.9-4.1)	13.2 (11.0-15.9)	21.5 (17.9-25.4)	46.2 (40.5-51.3)	60.7 (55.5-65.3)	85.6 (77.0-91.6)	91.4 (86.1-95.1)
Sierra Leone	29.0 (1.7-78.6)	37.4 (2.8-85.3)	46.5 (39.4-53.4)	59.1 (52.5-65.8)	81.9 (77.0-86.2)	88.3 (84.6-91.2)	91.8 (81.1-97.9)	94.8 (87.6-98.7)
Tajikistan	0.3 (0.0-1.8)	0.7 (0.0-5.0)	0.6 (0.1-2.2)	1.7 (0.2-6.2)	1.3 (0.5-2.5)	3.7 (1.6-7.3)	6.5 (0.2-29.1)	14.9 (0.7-58.6)
Tanzania	30.8 (0.8-90.7)	44.5 (1.9-95.9)	43.5 (17.7-72.4)	63.5 (35.3-85.8)	58.6 (38.1-77.6)	76.9 (59.8-89.4)	67.1 (15.6-97.8)	80.2 (28.8-99.0)
The Gambia	36.8 (3.0-88.6)	33.0 (2.2-86.4)	48.2 (35.6-63.4)	42.8 (30.1-57.3)	43.1 (34.5-51.7)	37.8 (30.7-45.3)	55.2 (18.0-90.3)	50.5 (14.3-88.1)
Timor-Leste	5.7 (0.2-32.3)	7.1 (0.3-41.2)	9.6 (7.0-12.8)	12.3 (9.1-15.9)	14.9 (4.9-31.6)	18.7 (6.2-37.5)	20.3 (0.8-71.2)	24.2 (1.0-75.5)
Togo	33.5 (0.2-96.0)	40.7 (0.5-97.6)	46 (25.8-67.2)	58.6 (38.0-78.2)	58.0 (25.6-86.0)	69.1 (39.0-91.1)	64.9 (9.1-98.4)	72.8 (16.0-99.0)
Uganda	15 (3.9-37.5)	18.3 (4.9-42.8)	44.1 (37.1-50.9)	50.4 (44.1-56.9)	65.9 (54.3-76.4)	71.3 (60.3-80.7)	77.8 (35.4-97.2)	81.4 (41.8-97.9)
Ukraine	1.5 (0.0-9.4)	4.3 (0.1-25.4)	4.8 (2.2-9.0)	13.2 (6.3-21.8)	12.7 (0.7-53.8)	26.3 (2.1-78.7)	24.9 (0.1-93.1)	38.7 (0.5-98.0)
Yemen	4.3 (0.0-34.5)	2.6 (0.0-21.8)	3.3 (1.1-7.4)	1.7 (0.6-3.6)	3.9 (0.1-19.5)	2.1 (0.0-11.4)	12.7 (0.1-70.3)	8.3 (0.0-54.5)

Zambia	29.2 (0.6-92.9)	40.7 (1.4-96.8)	32.7 (10.5-61.0)	50.9 (21.8-78.0)	68.4 (53.9-80.7)	83.1 (73.3-90.3)	85.2 (61.1-96.6)	92.7 (78.1-98.4)
Zimbabwe	1.0 (0.2-2.7)	0.5 (0.1-1.2)	2.0 (1.2-3.1)	0.9 (0.6-1.4)	7.8 (4.2-13.1)	3.8 (2.1-6.5)	30.8 (7.1-64.7)	18.3 (3.6-44.8)

DRC: Democratic Republic of the Congo, STP: Sao Tome and Principe

Table S4: Magnitude of wealth-based inequality in the coverage of deworming among pregnant women between 2000 and 2030

Country	Slope Index of Inequality				
	2000	2010	2020	2030	Difference*
Afghanistan	0.8	2.4	2.6	4.8	3.9
Albania	-0.3	-0.3	-0.2	-0.3	0.0
Angola	27.9	49.8	45.6	42.7	14.8
Armenia	0.5	1.0	3.9	17.9	17.4
Azerbaijan	0.5	6.7	8.1	10.3	9.8
Benin	34.8	38.6	50.1	47.4	12.6
Burkina Faso	2.6	6.7	7.0	5.6	3.1
Burundi	1.5	8.3	7.6	1.7	0.2
Cambodia	1.8	13.9	4.0	0.8	-1.0
Cameroon	25.5	35.1	28.4	29.4	3.9
Chad	6.3	13.2	14.7	14.2	7.9
Comoros	9.5	13.1	11.7	8.9	-0.6
Congo	33.1	30.3	30.9	21.3	-11.8
DRC	19.4	28.4	26.9	18.9	-0.5
Dominican Republic	-4.7	-6.1	-9.2	-10.5	-5.9
Egypt	0.2	0.1	0.1	0.3	0.1
Ethiopia	2.5	3.9	5.5	7.1	4.6
Gabon	19.9	26.2	24.2	17.0	-2.9
Ghana	-2.8	-7.2	-7.3	-7.1	-4.3
Guatemala	-0.7	-1.3	-3.0	-4.9	-4.2
Guinea	23.2	32.1	39.4	37.3	14.1
Guyana	-4.7	-8.2	-8.6	-5.2	-0.5
Haiti	7.2	7.9	4.3	6.3	-0.9
Honduras	-3.0	-2.0	-1.4	-1.1	2.0
India	1.3	8.1	17.2	21.3	20.1
Kenya	2.2	7.7	12.9	10.4	8.3
Kyrgyzstan	2.2	4.7	11.2	13.6	11.5
Liberia	7.7	14.4	13.8	8.2	0.5
Madagascar	4.9	6.7	4.8	3.8	-1.1
Malawi	0.4	0.5	0.7	0.6	0.2
Maldives	-7.5	-22.7	-30.1	-28.4	-21.0
Mali	6.6	11.9	17.5	13.9	7.3
Mozambique	24.1	31.5	32.0	28.2	4.2
Myanmar	3.4	2.5	6.2	4.9	1.5
Namibia	-3.8	-6.1	-2.9	-1.8	2.0
Nepal	1.2	4.5	3.3	0.9	-0.3

Niger	13.9	21.3	19.3	14.5	0.6
Nigeria	1.8	12.0	19.5	30.2	28.4
Pakistan	3.3	1.6	0.9	0.4	-2.8
Papua New Guinea	4.4	6.9	11.0	13.6	9.3
Peru	-0.4	-2.9	-2.0	-3.3	-2.9
Philippines	-2.6	-4.2	-4.6	-4.5	-1.9
Rwanda	0.4	5.0	5.5	4.8	4.5
STP	9.6	12.4	8.2	6.2	-3.5
Senegal	1.1	9.9	17.2	6.8	5.7
Sierra Leone	10.6	16.0	8.2	3.9	-6.7
Tajikistan	0.4	1.3	2.9	10.0	9.5
Tanzania	17.3	25.3	23.2	16.6	-0.7
The Gambia	-5.5	-7.9	-7.7	-6.8	-1.3
Timor-Leste	1.7	3.3	4.6	4.6	3.0
Togo	10.7	18.9	16.9	12.1	1.4
Uganda	4.3	8.5	7.4	4.8	0.4
Ukraine	3.4	10.0	16.0	16.1	12.7
Yemen	-2.5	-2.4	-2.7	-6.4	-3.9
Zambia	15.0	23.8	19.4	9.9	-5.1
Zimbabwe	-0.6	-1.3	-4.5	-14.2	-13.6

*The difference of slope index of inequality between 2000 and 2030

DRC: Democratic Republic of the Congo, STP: Sao Tome and Principe

Table S5: Posterior mean difference by considering with and without country level predictors for deworming coverage among pregnant women

Country	Posterior mean differences by changing predictors (with and without country level predictors)						
	**2000**	**2005**	**2010**	**2015**	**2020**	**2025**	**2030**
Afghanistan	1.0	0.5	0.0	0.0	0.2	0.9	1.8
Albania	0.4	0	0.0	0.0	0.0	-0.1	-0.1
Angola	-0.3	-0.2	-0.1	0.3	0.6	0.5	0.4
Armenia	0.1	0.0	0.0	0.0	-0.3	-1.5	-2.6
Azerbaijan	0.3	0.1	-0.4	-1.8	-3.3	-6.3	-8.7
Benin	-0.5	-0.1	-0.1	0.3	-0.6	-1.1	-1.5
Burkina Faso	1.6	0.8	-0.2	-1.0	-2.0	-2.1	-2.0
Burundi	-0.5	0.0	0.1	0.1	-0.2	-0.2	0.0
Cambodia	0.5	0.1	0.4	0.4	0.4	0.3	0.2
Cameroon	0.7	0.2	0.1	0.1	0.3	0.2	0.1
Chad	1.9	0.7	0.2	-0.1	-0.3	0.1	0.8
Comoros	-6.1	-4.7	-0.4	0.1	1.8	2.2	3.1
Congo	-5.1	-3.1	-0.5	0.2	1.3	2.1	2.6
DRC	7.3	7.2	3.3	-0.3	-1.1	-1.2	-2.6
Dominican Republic	1.4	1.8	0.1	0.1	-0.3	-0.2	-0.3
Egypt	0.8	0.6	0.3	0.0	-0.6	-2.2	-3.7
Ethiopia	-0.1	-0.7	-0.4	-0.3	0.2	1.0	1.8
Gabon	2.5	2.4	0.7	-0.6	-0.8	-0.4	-0.5
Ghana	4.1	2.6	-1.6	0.3	1.6	1.1	0.3
Guatemala	-0.1	-0.1	-0.1	-0.1	0.0	0.0	0.0
Guinea	3.2	3.1	1.4	0.6	0.5	2.4	4.9
Guyana	0.7	0.2	-0.4	-1.9	-2.6	-5.1	-5.6
Haiti	0.1	0.0	0.2	0.0	-0.3	-0.4	-1.1
Honduras	0.3	0.3	0.0	0.1	0.2	0.1	0.3
India	-0.1	0.0	-0.1	0.0	0.0	0.7	0.5
Kenya	0.1	0.1	0.0	0.0	0.5	1.0	1.3
Kyrgyzstan	0.7	0.5	0.1	-0.6	-2.6	-5.4	-7.9
Liberia	0.0	-0.1	0.2	0.5	0.4	0.7	0.7
Madagascar	2.2	0.6	-0.7	-3.0	-4.7	-5.4	-6.0
Malawi	1.6	1.1	0.0	-0.4	-0.4	0.0	-0.1
Maldives	2.2	1.3	0.3	-1.3	-3.5	-4.0	-4.9
Mali	2.7	1.7	0.0	0.0	0.3	0.9	1.2
Mozambique	-3.7	-4.9	-0.5	-2.6	-4.6	-3.4	-1.8
Myanmar	4.2	4.7	4.9	0.5	-1.2	-2.4	-2.9
Namibia	0.4	0.1	-0.1	0.1	0.4	0.3	0.5
Nepal	-0.2	-0.2	0.0	0.0	0.0	0.0	0.0

Niger	9.4	6.1	1.4	-3.3	-7.2	-9.8	-11.3
Nigeria	0.3	0.0	-0.1	-0.1	0.1	1.6	3.5
Pakistan	-0.6	-0.1	-0.1	-0.1	0.0	0.0	0.1
Papua New Guinea	4.4	3.4	2.2	0.4	-1.0	-3.0	-4.2
Peru	-0.1	-0.1	-0.1	-0.5	-0.8	-1.4	-2.3
Philippines	-0.1	0.0	-0.1	0.0	-0.1	0.0	0.0
Rwanda	-1.0	-1.5	0.8	0.4	-0.3	0.7	0.2
STP	-4.5	-1.4	1.6	5.8	7.6	8.5	9.0
Senegal	0.0	-0.1	0.0	-0.2	0.1	0.4	0.4
Sierra Leone	3.2	1.4	-0.1	0.3	-0.2	-0.5	-0.7
Tajikistan	0.5	0.3	0.2	0.1	-0.1	-0.5	-1.4
Tanzania	0.3	-1.3	-0.1	0.4	0.2	0.0	0.0
The Gambia	2.4	0.6	0.1	1.3	0.4	-0.5	-1.0
Timor-Leste	-1.4	-0.3	-0.2	-0.7	2.2	4.3	5.9
Togo	5.6	4.4	0.8	-0.7	-1.0	-1.5	-1.9
Uganda	2.7	0.5	-0.1	0.4	0.3	-0.2	0.0
Ukraine	1.0	0.2	-0.7	-0.6	-2.0	-3.6	-4.8
Yemen	-0.2	-0.1	-0.2	0.2	1.1	2.2	3.6
Zambia	10.8	7.2	-4.6	1.2	0.4	-1.2	-1.9
Zimbabwe	0.0	0.0	-0.1	-0.1	-0.2	-0.6	-1.8
All countries (median)	0.40	0.10	0.00	0.00	-0.05	-0.05	0.00
All countries (mean)	1.02	0.64	0.13	-0.11	-0.38	-0.57	-0.72

1 DRC: Democratic Republic of the Congo, STP: Sao Tome and Principe

Table S6: National level estimate of Gelman Rubin Potential scale reduction factors (PSRF) for deworming coverage among pregnant women

Country	Potential scale reduction factors (Point estimate; Upper CI)			
	2000	2010	2020	2030
Afghanistan	(1.004;1.004)	(1.004;1.004)	(1.004;1.004)	(1.004;1.004)
Albania	(1.004;1.004)	(1.004;1.004)	(1.004;1.004)	(1.004;1.004)
Angola	(1.004;1.004)	(1.004;1.004)	(1.004;1.004)	(1.004;1.004)
Armenia	(1.004;1.004)	(1.004;1.004)	(1.004;1.004)	(1.004;1.004)
Azerbaijan	(1.004;1.004)	(1.004;1.004)	(1.004;1.004)	(1.004;1.004)
Benin	(1.004;1.004)	(1.004;1.004)	(1.004;1.004)	(1.004;1.004)
Burkina Faso	(1.004;1.004)	(1.004;1.004)	(1.004;1.004)	(1.004;1.004)
Burundi	(1.004;1.004)	(1.004;1.004)	(1.004;1.004)	(1.004;1.004)
Cambodia	(1.004;1.004)	(1.004;1.004)	(1.004;1.004)	(1.004;1.004)
Cameroon	(1.004;1.004)	(1.004;1.004)	(1.004;1.004)	(1.004;1.004)
Chad	(1.004;1.004)	(1.004;1.004)	(1.004;1.004)	(1.004;1.004)
Comoros	(1.004;1.004)	(1.004;1.004)	(1.004;1.004)	(1.004;1.004)
Congo	(1.004;1.004)	(1.004;1.004)	(1.004;1.004)	(1.004;1.004)
DRC	(1.004;1.004)	(1.004;1.004)	(1.004;1.004)	(1.004;1.004)
Dominican Republic	(1.004;1.004)	(1.004;1.004)	(1.004;1.004)	(1.004;1.004)
Egypt	(1.004;1.004)	(1.004;1.004)	(1.004;1.004)	(1.004;1.004)
Ethiopia	(1.004;1.004)	(1.004;1.004)	(1.004;1.004)	(1.004;1.004)
Gabon	(1.004;1.004)	(1.004;1.004)	(1.004;1.004)	(1.004;1.004)
Ghana	(1.004;1.004)	(1.004;1.004)	(1.004;1.004)	(1.004;1.004)
Guatemala	(1.004;1.004)	(1.004;1.004)	(1.004;1.004)	(1.004;1.004)
Guinea	(1.004;1.004)	(1.004;1.004)	(1.004;1.004)	(1.004;1.004)
Guyana	(1.004;1.004)	(1.004;1.004)	(1.004;1.004)	(1.004;1.004)
Haiti	(1.004;1.004)	(1.004;1.004)	(1.004;1.004)	(1.004;1.004)
Honduras	(1.004;1.004)	(1.004;1.004)	(1.004;1.004)	(1.004;1.004)
India	(1.004;1.004)	(1.004;1.004)	(1.004;1.004)	(1.004;1.004)
Kenya	(1.004;1.004)	(1.004;1.004)	(1.004;1.004)	(1.004;1.004)
Kyrgyzstan	(1.004;1.004)	(1.004;1.004)	(1.004;1.004)	(1.004;1.004)
Liberia	(1.004;1.004)	(1.004;1.004)	(1.004;1.004)	(1.004;1.004)
Madagascar	(1.004;1.004)	(1.004;1.004)	(1.004;1.004)	(1.004;1.004)
Malawi	(1.004;1.004)	(1.004;1.004)	(1.004;1.004)	(1.004;1.004)
Maldives	(1.004;1.004)	(1.004;1.004)	(1.004;1.004)	(1.004;1.004)
Mali	(1.004;1.004)	(1.004;1.004)	(1.004;1.004)	(1.004;1.004)
Mozambique	(1.004;1.004)	(1.004;1.004)	(1.004;1.004)	(1.004;1.004)
Myanmar	(1.004;1.004)	(1.004;1.004)	(1.004;1.004)	(1.004;1.004)
Namibia	(1.004;1.004)	(1.004;1.004)	(1.004;1.004)	(1.004;1.004)
Nepal	(1.004;1.004)	(1.004;1.004)	(1.004;1.004)	(1.004;1.004)

Niger	(1.004;1.004)	(1.004;1.004)	(1.004;1.004)	(1.004;1.004)
Nigeria	(1.004;1.004)	(1.004;1.004)	(1.004;1.004)	(1.004;1.004)
Pakistan	(1.004;1.004)	(1.004;1.004)	(1.004;1.004)	(1.004;1.004)
Papua New Guinea	(1.004;1.004)	(1.004;1.004)	(1.004;1.004)	(1.004;1.004)
Peru	(1.004;1.004)	(1.004;1.004)	(1.004;1.004)	(1.004;1.004)
Philippines	(1.004;1.004)	(1.004;1.004)	(1.004;1.004)	(1.004;1.004)
Rwanda	(1.004;1.004)	(1.004;1.004)	(1.004;1.004)	(1.004;1.004)
STP	(1.004;1.004)	(1.004;1.004)	(1.004;1.004)	(1.004;1.004)
Senegal	(1.004;1.004)	(1.004;1.004)	(1.004;1.004)	(1.004;1.004)
Sierra Leone	(1.004;1.004)	(1.004;1.004)	(1.004;1.004)	(1.004;1.004)
Tajikistan	(1.004;1.004)	(1.004;1.004)	(1.004;1.004)	(1.004;1.004)
Tanzania	(1.004;1.004)	(1.004;1.004)	(1.004;1.004)	(1.004;1.004)
The Gambia	(1.004;1.004)	(1.004;1.004)	(1.004;1.004)	(1.004;1.004)
Timor-Leste	(1.004;1.004)	(1.004;1.004)	(1.004;1.004)	(1.004;1.004)
Togo	(1.004;1.004)	(1.004;1.004)	(1.004;1.004)	(1.004;1.004)
Uganda	(1.004;1.004)	(1.004;1.004)	(1.004;1.004)	(1.004;1.004)
Ukraine	(1.004;1.004)	(1.004;1.004)	(1.004;1.004)	(1.004;1.004)
Yemen	(1.004;1.004)	(1.004;1.004)	(1.004;1.004)	(1.004;1.004)
Zambia	(1.004;1.004)	(1.004;1.004)	(1.004;1.004)	(1.004;1.004)
Zimbabwe	(1.004;1.004)	(1.004;1.004)	(1.004;1.004)	(1.004;1.004)

CI: Confidence Intervals, DRC: Democratic Republic of the Congo, STP: Sao Tome and Principe

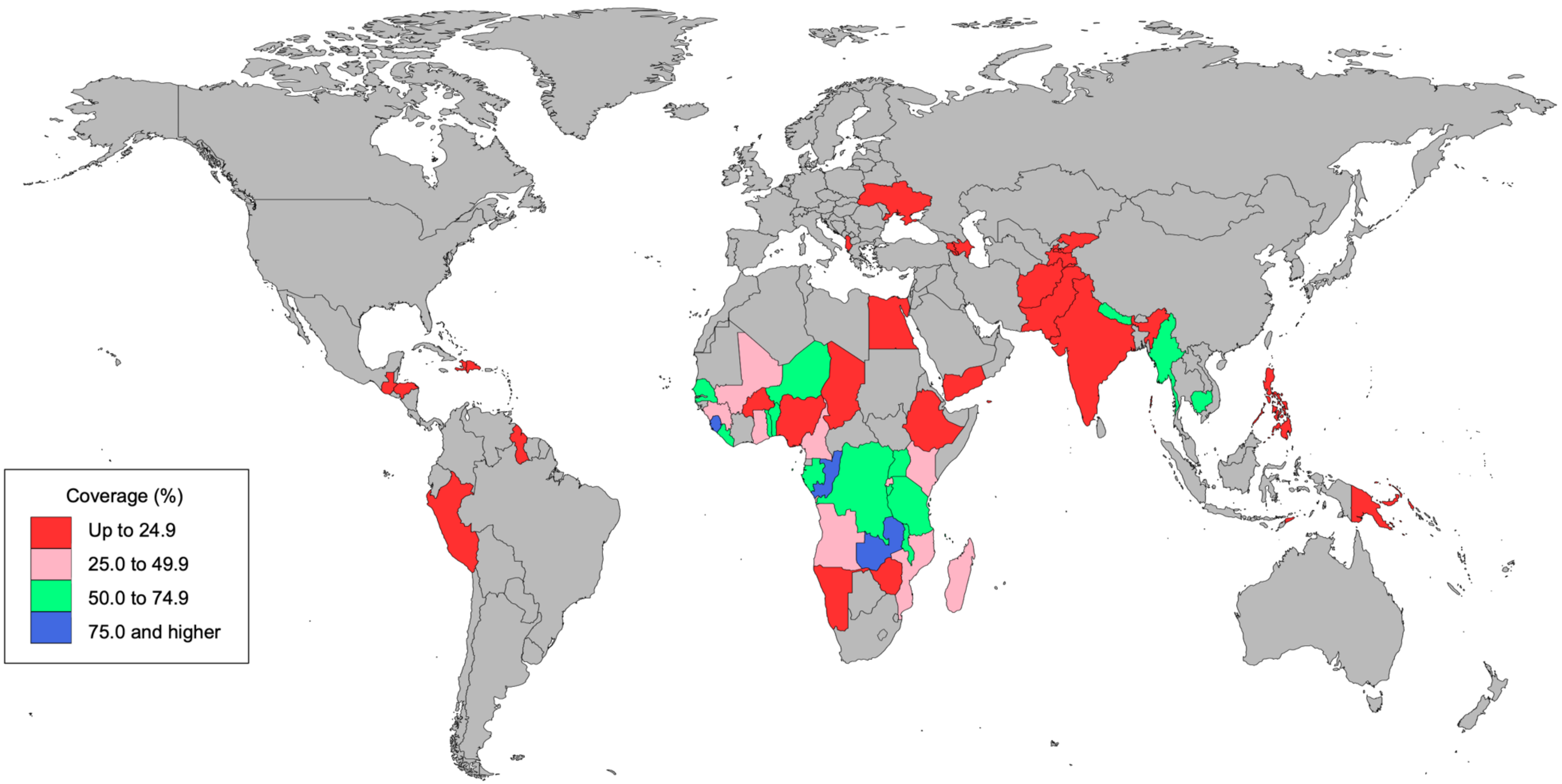

Figure S1: Geographical distribution and deworming coverage (%) heatmap based on the latest Demographic Health Survey data of included countries

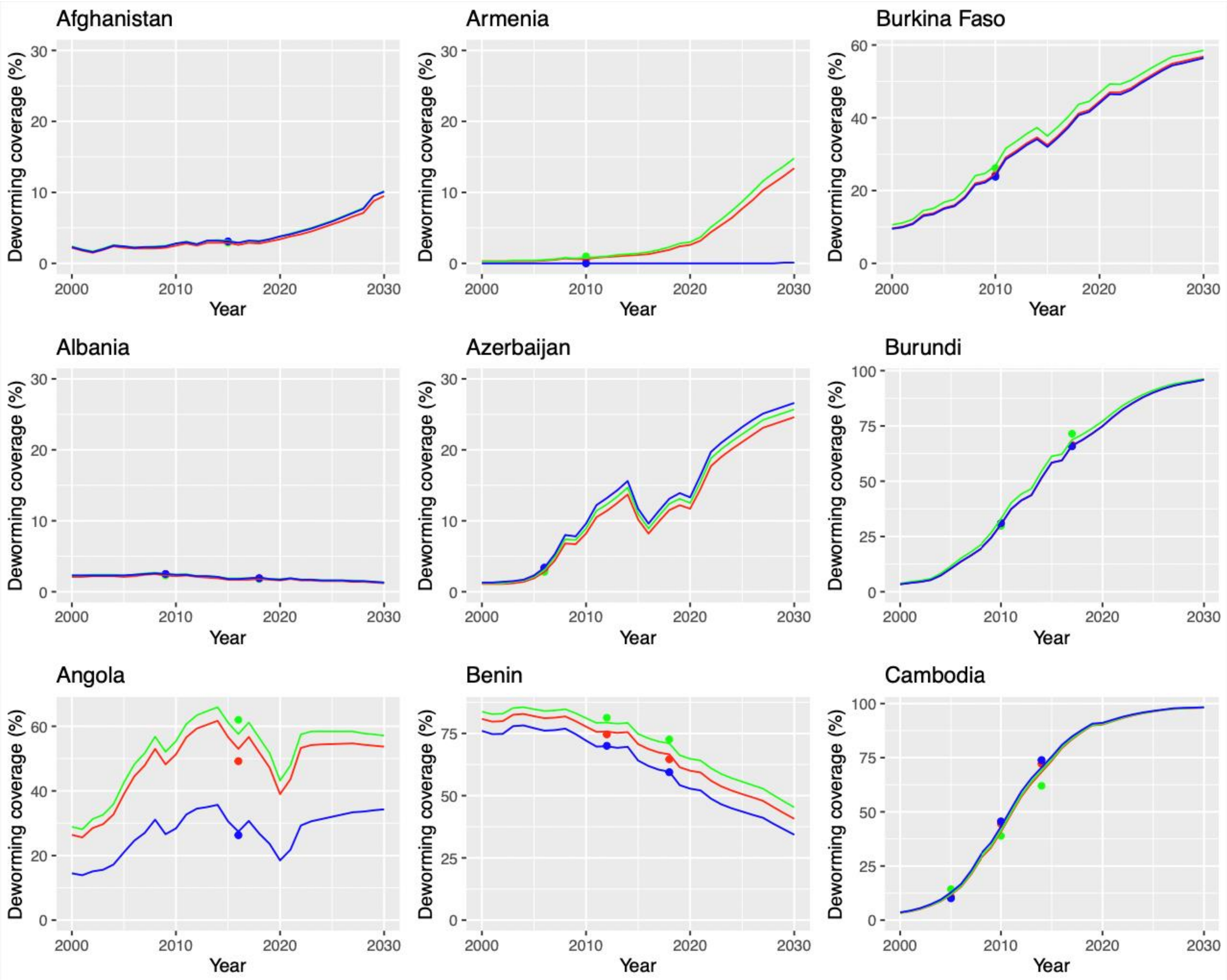
Afghanistan
Armenia
Burkina Faso
Albania
Azerbaijan
Burundi
Angola
Benin
Cambodia
Deworming coverage (%)
Year
2000
2010
2020
2030

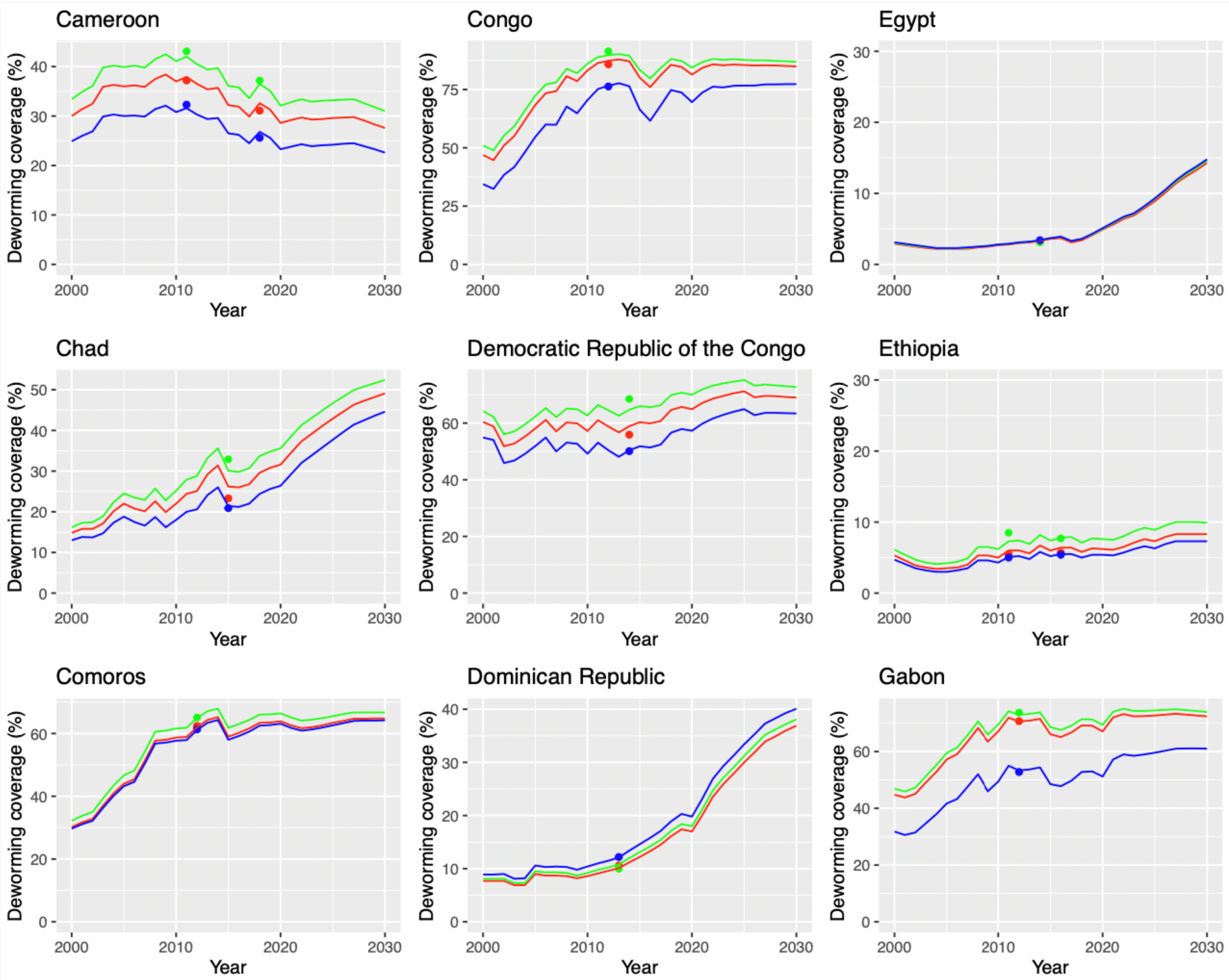
Cameroon
Congo
Egypt
Chad
Democratic Republic of the Congo
Ethiopia
Comoros
Dominican Republic
Gabon
Deworming coverage (%)
Year

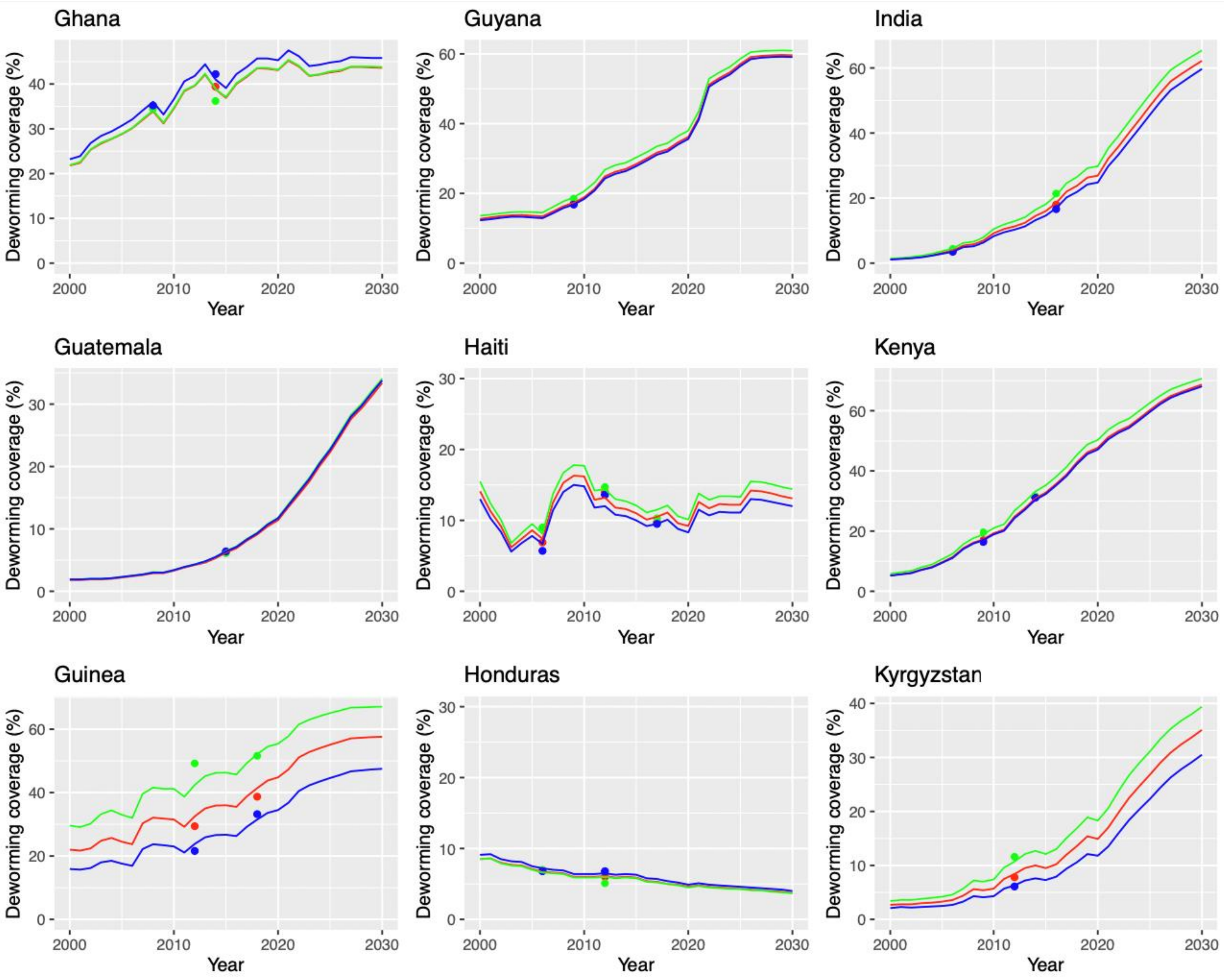
Ghana
Guyana
India
Guatemala
Haiti
Kenya
Guinea
Honduras
Kyrgyzstan
Deworming coverage (%)
Year
2000
2010
2020
2030

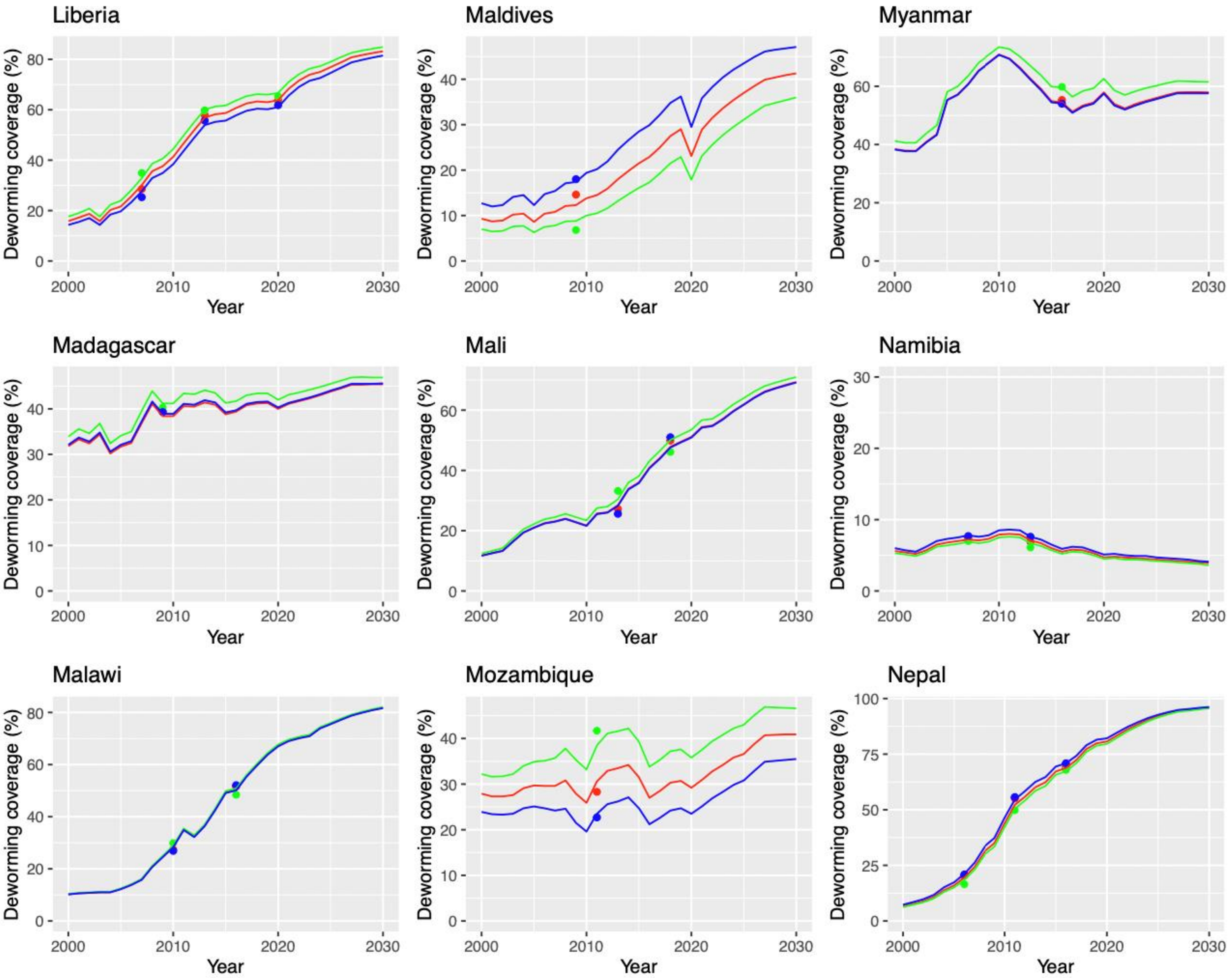
Liberia
Maldives
Myanmar
Madagascar
Mali
Namibia
Malawi
Mozambique
Nepal
Deworming coverage (%)
Year

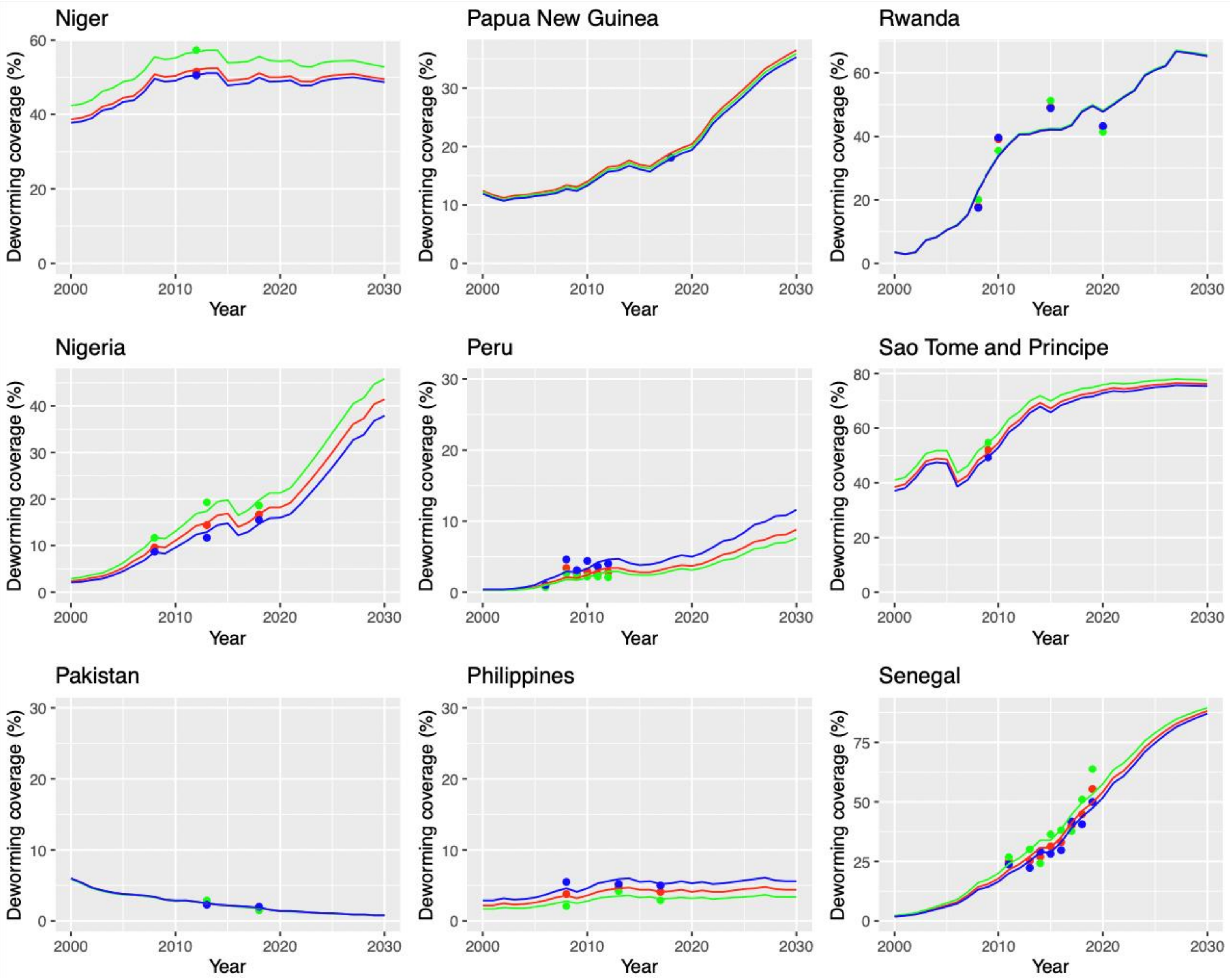
Niger
Papua New Guinea
Rwanda
Nigeria
Peru
Sao Tome and Principe
Pakistan
Philippines
Senegal
Deworming coverage (%)
Year
2000
2010
2020
2030

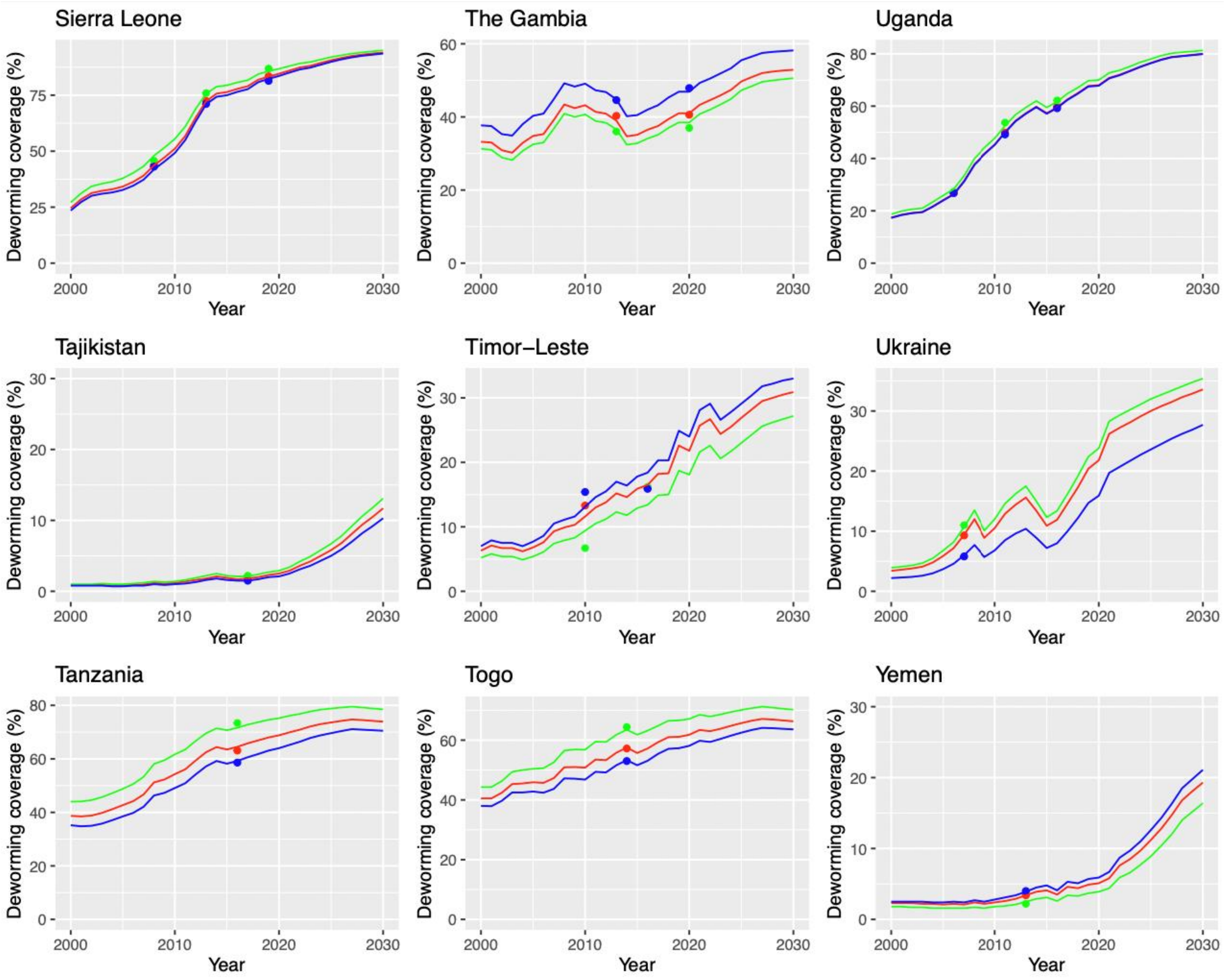
Sierra Leone
The Gambia
Uganda
Tajikistan
Timor−Leste
Ukraine
Tanzania
Togo
Yemen
Deworming coverage (%)
Year
2000
2010
2020
2030

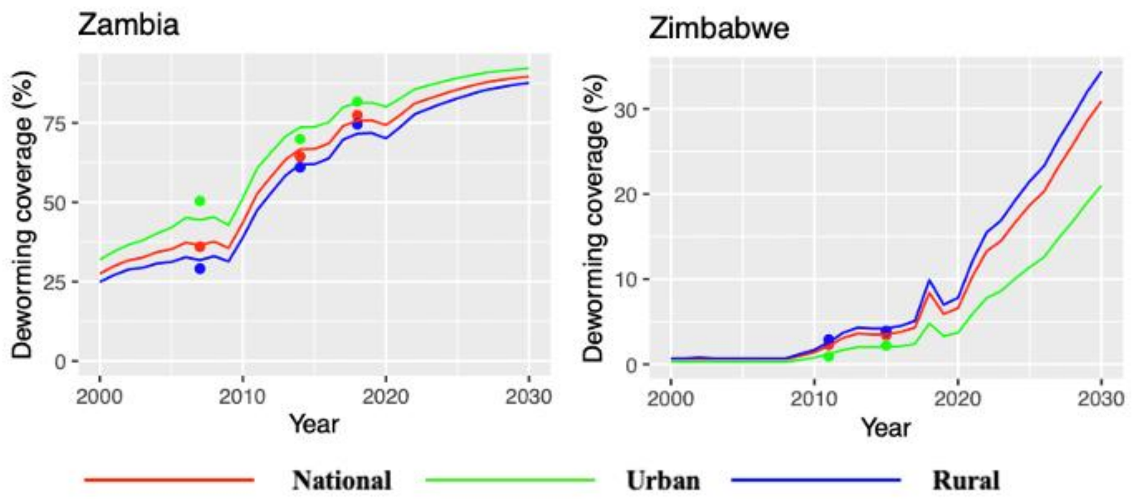

Figure S2: Detailed year-specific observed and predicted coverage of deworming among pregnant women by place of residence

Note: Deworming coverage from DHS survey data are plotted with dots and lines were drawn with projected values

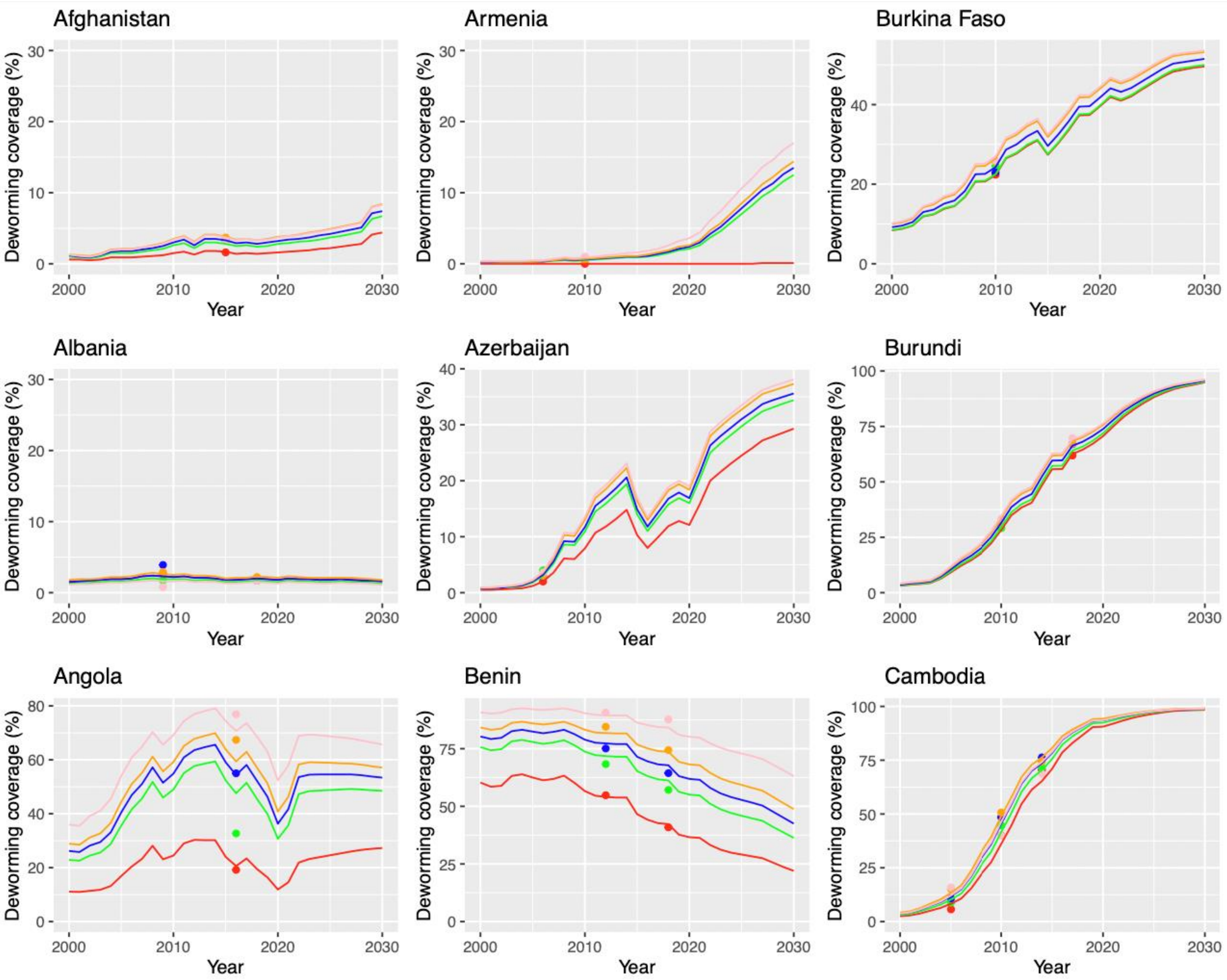

Afghanistan
Armenia
Burkina Faso
Albania
Azerbaijan
Burundi
Angola
Benin
Cambodia
Deworming coverage (%)
Year
2000
2010
2020
2030

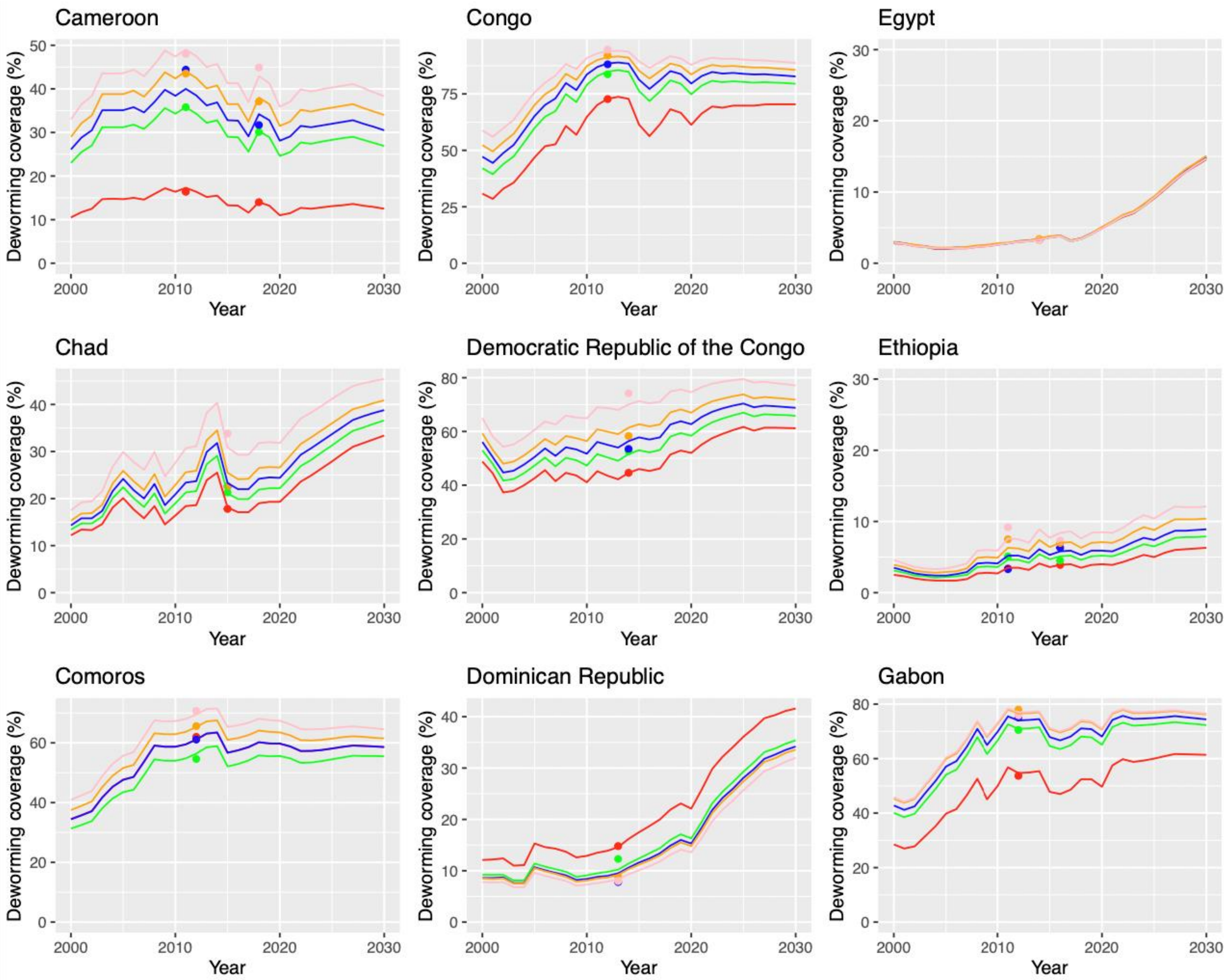

Cameroon
Congo
Egypt
Chad
Democratic Republic of the Congo
Ethiopia
Comoros
Dominican Republic
Gabon
Deworming coverage (%)
Year
2000
2010
2020
2030

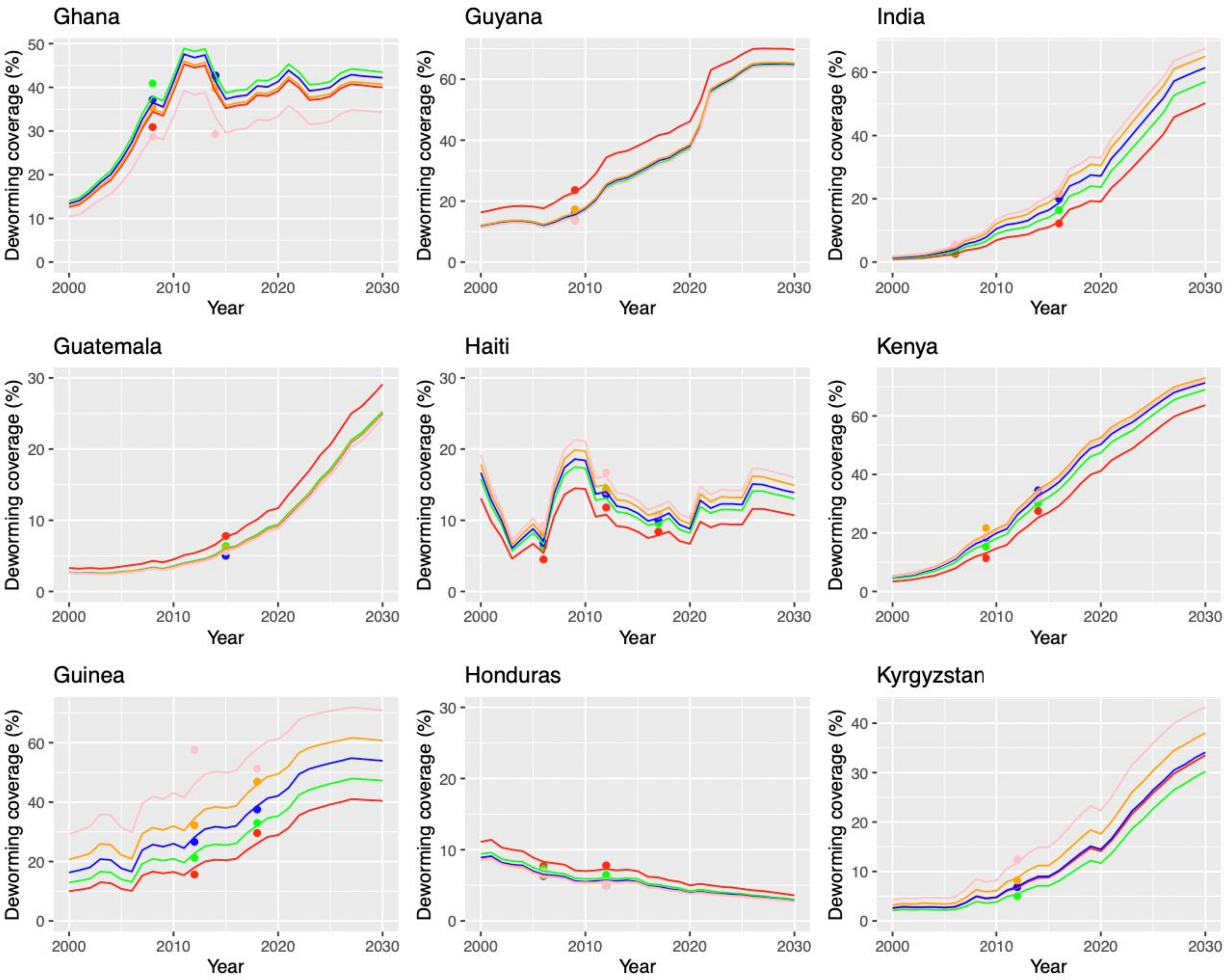

Ghana
Guyana
India
Guatemala
Haiti
Kenya
Guinea
Honduras
Kyrgyzstan
Deworming coverage (%)
Year

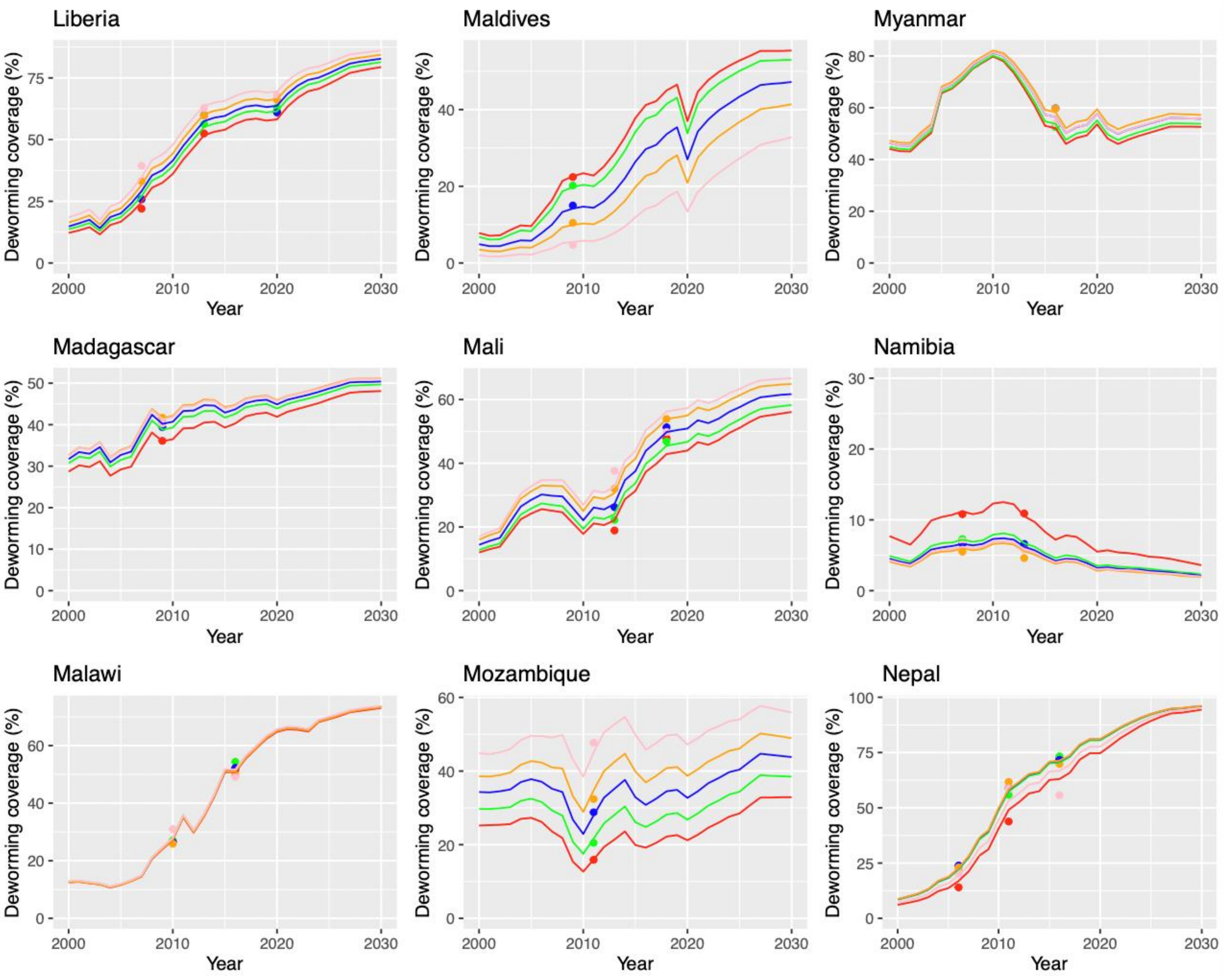
Liberia
Maldives
Myanmar
Madagascar
Mali
Namibia
Malawi
Mozambique
Nepal
Deworming coverage (%)
Year

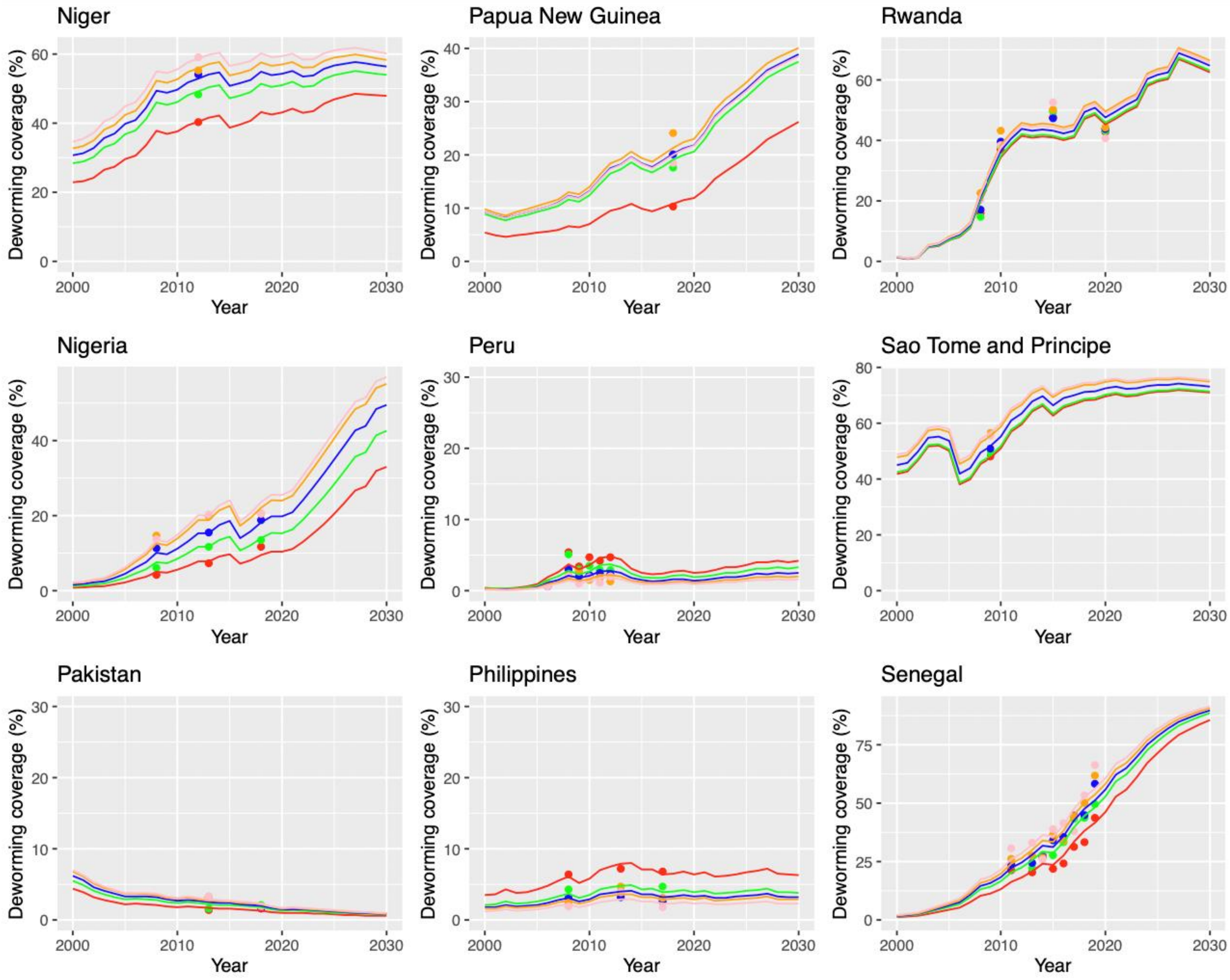
Niger
Papua New Guinea
Rwanda
Nigeria
Peru
Sao Tome and Principe
Pakistan
Philippines
Senegal
Deworming coverage (%)
Year
2000
2010
2020
2030

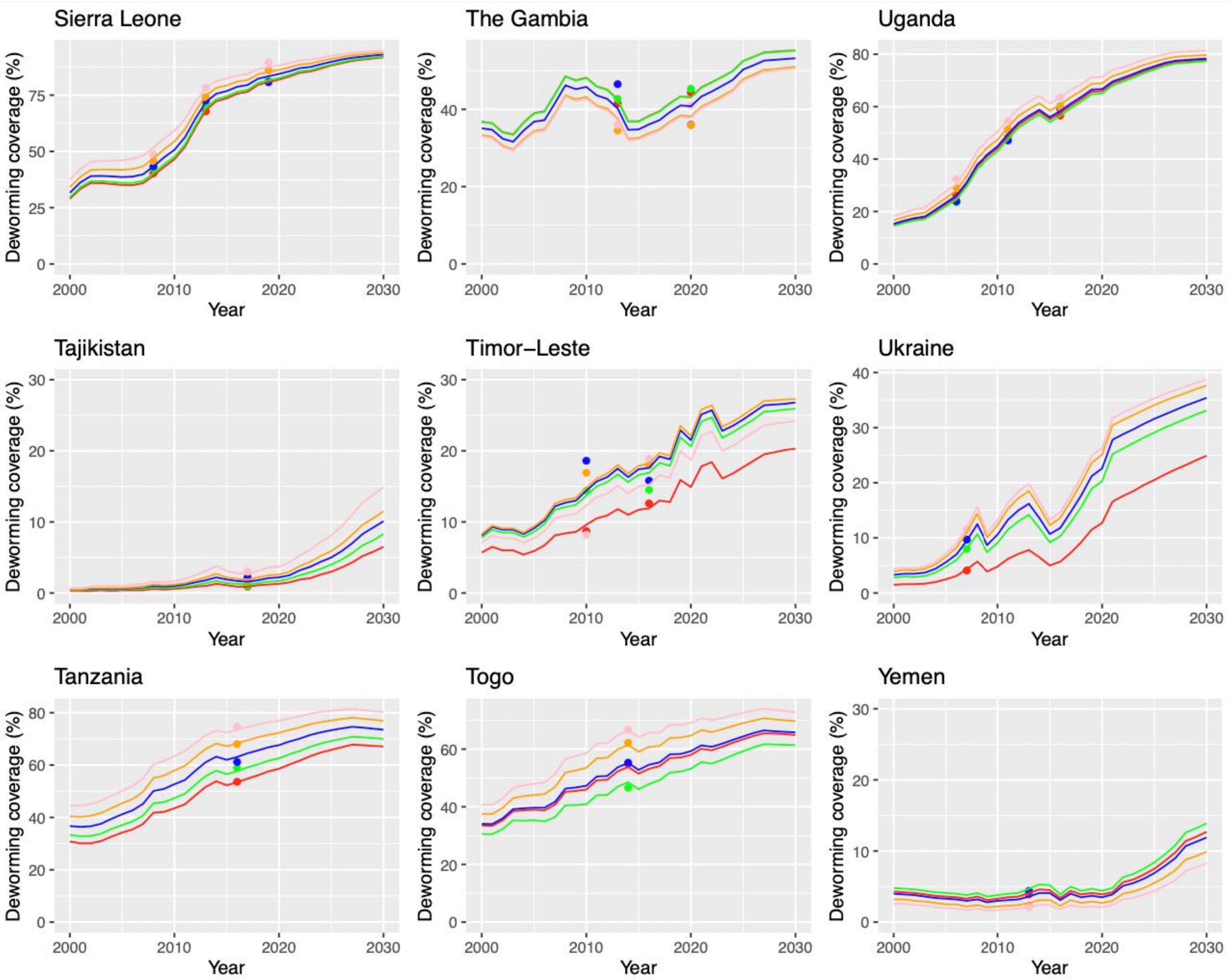
Sierra Leone
The Gambia
Uganda
Tajikistan
Timor−Leste
Ukraine
Tanzania
Togo
Yemen
Deworming coverage (%)
Year
2000
2010
2020
2030

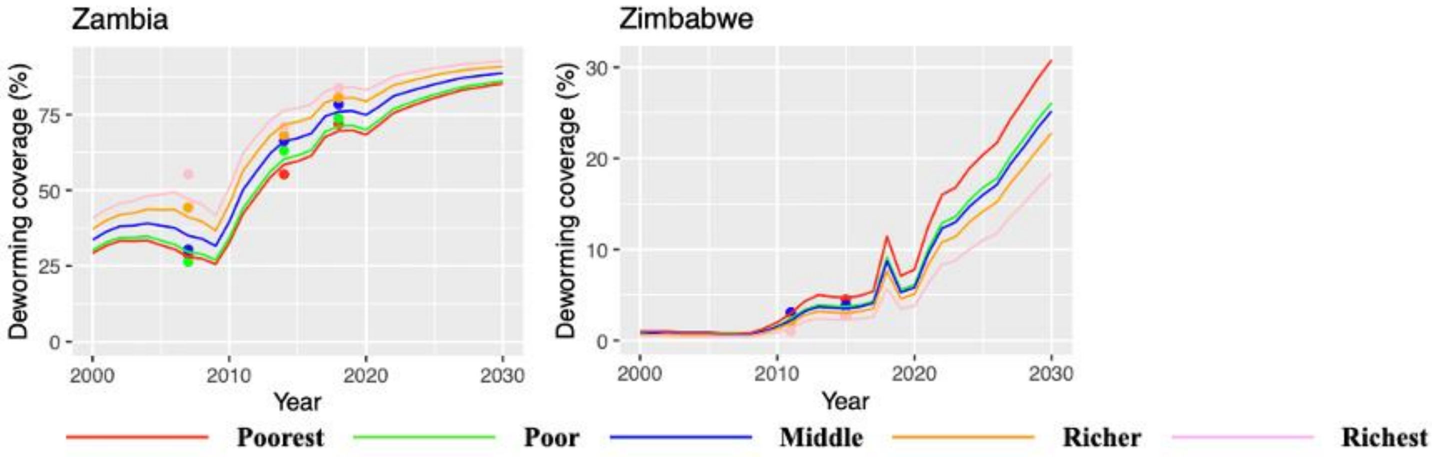

Figure S3: Detailed year-specific observed and predicted coverage of deworming among pregnant women by wealth quintile.

Note: Deworming coverage from DHS survey data are plotted with dots and lines were drawn with projected values
